# The Role and Mechanism of G Protein Subunit Alpha-15 in Colorectal Cancer: An Analysis of Two Hundred Eight Patient Samples and Public Datasets

**DOI:** 10.14740/wjon2647

**Published:** 2025-12-17

**Authors:** Da Tong Zeng, Zu Yuan Chen, Zuo Yan Ming Li, Bang Teng Chi, Jian Di Li, Wei Zhang, Zong Yu Li, Jian Jun Li, Hui Li, Rong Quan He, Gang Chen, Jia Ying Wen, Wei Jian Huang

**Affiliations:** a Department of Pathology, Redcross Hospital of Yulin City, Yulin 537000, Guangxi Zhuang Autonomous Region, China; b Department of General Surgery, The Second Affiliated Hospital of Guangxi Medical University, Nanning 530021, Guangxi Zhuang Autonomous Region, China; c Department of Pathology, The First Affiliated Hospital of Guangxi Medical University, Nanning 530021, Guangxi Zhuang Autonomous Region, China; d Department of Colorectal and Anal Surgery, The First Affiliated Hospital of Guangxi Medical University, Nanning 530021, Guangxi Zhuang Autonomous Region, China; e Department of Medical Oncology, The First Affiliated Hospital of Guangxi Medical University, Nanning 530021, Guangxi Zhuang Autonomous Region, China; f Department of Radiation Oncology, The Second Affiliated Hospital of Guangxi Medical University, Nanning 530021, Guangxi Zhuang Autonomous Region, China; g These authors contributed equally to this work.

**Keywords:** G protein subunit α-15, Colorectal cancer, Immunohistochemistry, Single-cell sequencing, Pathology, Tumor microenvironment

## Abstract

**Background:**

Colorectal cancer (CRC) is a tumor with a relatively high incidence rate. The expression of G protein subunit α-15 (GNA15) in CRC and its specific role remain unclear.

**Methods:**

This study focused on both the diagnostic potential of GNA15 and its mechanistic role in CRC progression. Relevant expression data of CRC were obtained from global databases, and the expression differences of GNA15 in CRC tissues and non-cancerous tissues were analyzed after processing. Tissue microarrays of CRC samples and surrounding non-tumor tissues from 208 CRC patients at the Red Cross Hospital of Yulin City were collected and prepared. The expression level of GNA15 was scored after immunostaining with rabbit anti-human GNA15 antibody. Meanwhile, the *GNA15* gene was knocked out in CRC cells, and its effects were evaluated. Data from databases were used to explore the relationship between GNA15 expression and various pathways through Gene Set Enrichment Analysis (GSEA). Finally, the reliability of the conclusions was tested through statistical analysis.

**Results:**

The analysis results from the database and the tissue microarrays from the Red Cross Hospital of Yulin City both indicated that GNA15 was significantly overexpressed in CRC tissues. The gene effect scores calculated after knocking out GNA15 showed that the gene effect scores of HCC56 and DLD1 cell lines were strongly negative. GSEA indicated that the differentially expressed genes were mainly concentrated in biological processes such as antigen-antibody binding, immunoglobulin complex, phagocytosis, antigen processing and presentation, cell adhesion molecules, T helper 17 (Th17) cell differentiation, and toll-like receptor signaling pathway. This study has limitations, including a single-center retrospective cohort design and lack of *in vivo* validation.

**Conclusions:**

GNA15 is highly expressed in CRC and has certain diagnostic value. GNA15 may play a role in CRC through affecting the growth of cell lines such as HCC56, DLD1 and biological processes such as antigen-antibody binding, immunoglobulin complex, phagocytosis, antigen processing and presentation, cell adhesion molecules, Th17 cell differentiation, and toll-like receptor signaling pathway.

## Introduction

Colorectal cancer (CRC) is now the third most common cancer globally and the second leading cause of cancer death. Incidence has increased in recent years. According to projections, the global burden of CRC is expected to increase by 72% from 1.8 million new cases in 2018 to more than 3 million by 2040, with substantial increases expected in low - and middle-income countries [1, 2]. At present, a variety of targeted drugs such as epidermal growth factor receptor (EGFR) inhibitors (cetuximab, panitumumab) and vascular endothelial growth factor (VEGF) inhibitors (bevacizumab) have been used in the treatment of CRC, but the current targeted drug therapy still has problems of resistance and side effects. Some patients still receive poor benefits from targeted drug therapy now. Therefore, it is of great significance to search for new and potential therapeutic targets.

G protein subunit α-15 (GNA15) as the α subunit of G protein, its key role in cell signaling has made it an important target for biomedical research. Existing studies have confirmed that high expression of GNA15 is associated with poor prognosis in some malignancies, such as pancreatic ductal adenocarcinoma [3], adult acute myeloid leukemia [4], small intestinal neuroendocrine neoplasia [5] and serous ovarian cancer [6]. The exact mechanism by which GNA15 promotes tumorigenesis is not well understood. Existing studies suggest that GNA15 may induce malignant cell proliferation mainly by inhibiting apoptosis. GNA15 was proved to be able to couple with β1 adrenergic receptor and regulate proliferation signal through G protein-coupled receptor. When GNA15 expression was low, proliferation was inhibited and apoptosis was activated [5]. Some researchers treat cells *in vitro* with the monoclonal antibody (mAb) AC10364 and found that it could reduce the expression of GNA15 and induce apoptosis [7]. These studies all suggest that the high expression of GNA15 in tumor cells may lead to the inhibition of apoptosis and the development of tumors. In CRC, it remains unclear whether the carcinogenic effects of GNA15 are different from those in other tumors and whether it is a promising target for screening and prognostic determination.

Using data from Gene Expression Omnibus (GEO), Tumor Immune Estimation Resource (TIMER), and relevant databases, along with clinical data from 208 CRC patients at the Red Cross Hospital of Yulin City, this study clarified the expression differences of GNA15 in CRC cells and evaluated GNA15 expression through single-cell analysis. The correlation between GNA15 expression and tumor microenvironment (TME) was studied. At the same time, GNA15 was knocked out using the CRISPR-Cas9 system, and the gene effect score was calculated with the CERES (CRISPR Efficiency through expression level Regularization and Evaluation of Scores) algorithm to evaluate the role of GNA15 in the regulation of CRC cell proliferation. Through the above research, this study integrated multi-source data (public databases + 208 clinical samples) to clarify two core objectives: 1) the diagnostic value of GNA15 in CRC; 2) the mechanistic role of GNA15 in CRC progression via immune-related pathways.

## Materials and Methods

### Evaluation of differential expressions of GNA15 in CRC

#### Assessment of differential expressions of GNA15 mRNA in CRC based on global public databases

The expression profiles of GNA15 in CRC were analyzed using data from the GEO, The Cancer Genome Atlas (TCGA), the Genotype-Tissue Expression (GTEx) project, the International Cancer Genome Consortium (ICGC), ArrayExpress, the Sequence Read Archive (SRA), and relevant scientific literature. The search keywords included “colon,” “rectal,” “rectum,” “colorectal,” “carcinoma,” “cancer,” “malignancy,” and “tumor.” The inclusion criteria were as follows: 1) data derived from human samples; 2) experimental groups consisting of CRC tissue samples, with control groups comprising non-tumor tissues; 3) a minimum of three samples in each group. The exclusion criteria were: 1) samples from recurrent or metastatic CRC; 2) absence of GNA15 expression data. Following the screening process, datasets from the same GEO platform were consolidated to form a comprehensive expression matrix. The mRNA expression matrix was standardized and log_2_ (x + 1) transformed, with batch effects removed using the “limma” and “sva” packages in R.

#### Immunohistochemical validation of GNA15 protein expression in CRC

This study utilized a tissue microarray containing 208 pairs of CRC and corresponding non-tumor tissue samples from the Red Cross Hospital of Yulin City to elucidate the expression of GNA15 protein in CRC. We collected clinical pathological data from the patients, including age, gender, macroscopic subtype, vascular invasion, neural invasion, lymph node involvement, TNM staging, survival status, and clinical stage (Supplementary Material 1, wjon.elmerpub.com). Institutional Review Board approval for this study was obtained from the Ethics Committees of both the Red Cross Hospital of Yulin City and the First Affiliated Hospital of Guangxi Medical University. This research was conducted in accordance with the ethical standards for human subjects set by the responsible institution.

During tissue analysis, paraffin-embedded blocks were cut into 2-µm thick sections and heated at 75 °C for 20 min to remove the paraffin, thereby facilitating histological examination. After deparaffinization, sections were incubated in 3% hydrogen peroxide for 15 min, followed by multiple washes with distilled water and phosphate-buffered saline (PBS). We performed immunostaining using rabbit anti-human GNA15 antibody (AB225949), followed by washing with PBS. Subsequently, the sections underwent re-staining, dehydration, clearing, and mounting. Adjacent non-cancerous tissue sections served as positive controls, while PBS was used as a negative control. The immunohistochemical staining was analyzed to compare the expression between CRC and adjacent non-cancerous tissues, with the collected data being synthesized.

Two experienced pathologists independently evaluated the processed sections. The evaluation criteria included the percentage of positively stained cells and staining intensity. The scoring system for the percentage of positively stained cells was as follows: 0 points for no expression, 1 point for fewer than 25% positive cells, 2 points for 26% to 50% positive cells, 3 points for 51% to 75% positive cells, and 4 points for over 75% positive cells. The staining intensity was scored as 0 for unstained, 1 for weak cytoplasmic staining, 2 for moderate yellow staining, and 3 for strong brown-yellow staining [8-10]. The overall score was derived by multiplying the staining intensity by the percentage of positively stained cells. In cases where the scores from the two pathologists differed, the average of their scores was taken as the final score.

#### Evaluation of GNA15 expression in single-cell analyses of CRC

This study employed single-cell RNA sequencing data from the GEO dataset GSE144735 to investigate the single-cell expression patterns of the *GNA15* gene in CRC. The criteria for cell selection included a total RNA feature count ranging from 200 to 2,500, and a requirement for mitochondrial DNA content to be below 5%. Data analysis was performed using the Seurat package. During this process, the data were normalized using the NormalizeData function. To address batch effects arising from differences in sequencing technologies, the Harmony package was utilized for integration. Subsequently, principal component analysis (PCA) was conducted using the FindVariableFeatures function, with a resolution parameter set to 0.5. Dimensionality reduction was achieved using Uniform Manifold Approximation and Projection (UMAP), with the analysis range set from 1 to 20 dimensions. GNA15 expression in normal and malignant epithelial cells of colorectal tissue was illustrated using violin plots.

### Correlation between GNA15 expression and the TME

The TIMER database serves as an online tool focused on the tumor immune microenvironment, aimed at providing researchers with in-depth analyses of immune cell infiltration in tumor patients [11]. To clarify the immune infiltration status of GNA15 in CRC, we downloaded data for seven immune cell types, including purity, B cells, CD8^+^ T cells, CD4^+^ T cells, macrophages, neutrophils, and dendritic cells.

### Investigation of the role of GNA15 in regulating CRC cell proliferation

In this segment of the study, we explored the functional role of GNA15 in CRC cells by elimination of GNA15 using CRISPR-Cas9 gene editing technology. CRISPR-Cas9 knockout data were retrieved from the DepMap public database, and gene effect scores were calculated via the CERES algorithm (details of Research Resource Identifiers (RRIDs) are provided in Table 1). The CERES algorithm was utilized to compute gene effect scores to assess the importance of GNA15 across various CRC cell lines. A negative gene effect score indicated that the knockout of GNA15 resulted in a significant disruption of cell growth, underscoring its essential role in cell proliferation [12].

**Table 1:** DepMap CRISPR-Cas9 Knockout Gene Effect Scores

Cell line name	DepMap ID	Gene effect score
HCC56	ACH-000467	-0.24839
DLD1	ACH-001061	-0.22831
CL40	ACH-000798	-0.19379
JVE127	ACH-002659	-0.19327
SNU1033	ACH-000286	-0.19194
COLO678	ACH-000350	-0.18189
SNUC1	ACH-000722	-0.17757
CCK81	ACH-000963	-0.15942
KP363T	ACH-002669	-0.15636
RKO	ACH-000943	-0.14606
T84	ACH-000381	-0.14006
NCIH747	ACH-000403	-0.13433
C2BBE1	ACH-000009	-0.13306
JVE015	ACH-002654	-0.11808
SNU503	ACH-000683	-0.11213
LS411N	ACH-000985	-0.1076
SW1116	ACH-000489	-0.08493
SW626	ACH-001399	-0.08292
LOVO	ACH-000950	-0.07995
SW837	ACH-000421	-0.07115
TT1TKB	ACH-002025	-0.06795
SW620	ACH-000651	-0.0569
NCIH716	ACH-000491	-0.04153
HCC2998	ACH-001081	-0.04031
COLO201	ACH-000253	-0.03847
KM12	ACH-000969	-0.03499
LS180	ACH-000957	-0.0335
HT115	ACH-000986	-0.02879
SNU61	ACH-000532	-0.02814
LS513	ACH-000007	-0.02252
JVE253	ACH-002664	-0.00896
MDST8	ACH-000935	-0.00034

ID: identifier.

### Potential pathogenic mechanisms of GNA15 in CRC

We obtained RNA sequencing data for CRC along with corresponding clinical information from TCGA database. For the genes involved in relevant pathways, we utilized the Gene Set Enrichment Analysis (GSEA) package in R for analysis. Spearman correlation analysis was performed to evaluate the relationship between gene expression and pathway scores.

### Statistical analysis

In the analysis of CRC and its non-cancerous colorectal tissues, we implemented a comprehensive assessment strategy that integrated mRNA microarray and sequencing data to illustrate the expression of the *GNA15* gene in CRC. The Wilcoxon test was performed using SPSS 22.0 to analyze the expression differences of GNA15 between CRC and non-tumor tissues. Significant differences were visualized using the ggplot2 package in R software, and standardized mean differences (SMDs) along with their 95% confidence intervals (CIs) were calculated in STATA 12.0. Model selection was based on heterogeneity analysis (I^2^ value and Chi-squared test); a random-effects model was employed in cases of significant heterogeneity (I^2^ > 50% and P < 0. 05). Furthermore, we assessed the expression differences of GNA15 between CRC and non-cancerous colorectal tissues using non-parametric tests, with a two-tailed P value of < 0. 05 considered statistically significant. We constructed receiver operating characteristic (ROC) curves using the pROC package in R and calculated the summary ROC (SROC) curves to evaluate the expression of GNA15 between CRC tissues and non-cancerous colorectal tissues, assessing its performance through the area under the curve (AUC). Additionally, we evaluated the characteristics of GNA15 using sensitivity, specificity, and diagnostic likelihood ratio (DLR). Publication bias was assessed using Egger’s and Begg’s tests, with a P ≥ 0. 05 indicating no significant publication bias.

## Results

### GNA15 is highly expressed in CRC

#### GNA15 protein is highly expressed in CRC according to immunohistochemical validation

**Figure 1:**
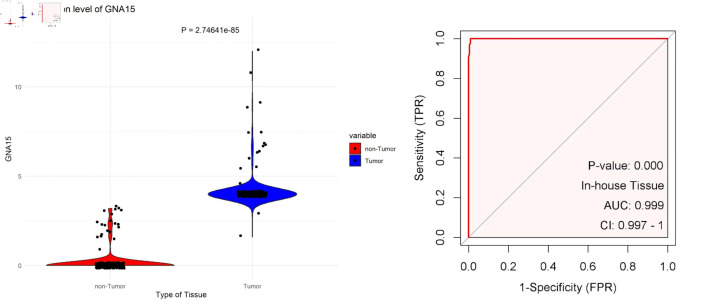
The left plot uses scatter-violin visualization to show that GNA15 expression is significantly higher in tumor (blue) than non-tumor (red) tissues. The right ROC curve, based on in-house tissue samples, shows that GNA15 has excellent diagnostic ability to distinguish tumor from non-tumor tissues. GNA15: G protein subunit α-15; ROC: receiver operating characteristic; AUC: area under the curve; FPR: false positive rate; CI: confidence interval; TPR: true positive rate.

**Figure 2:**
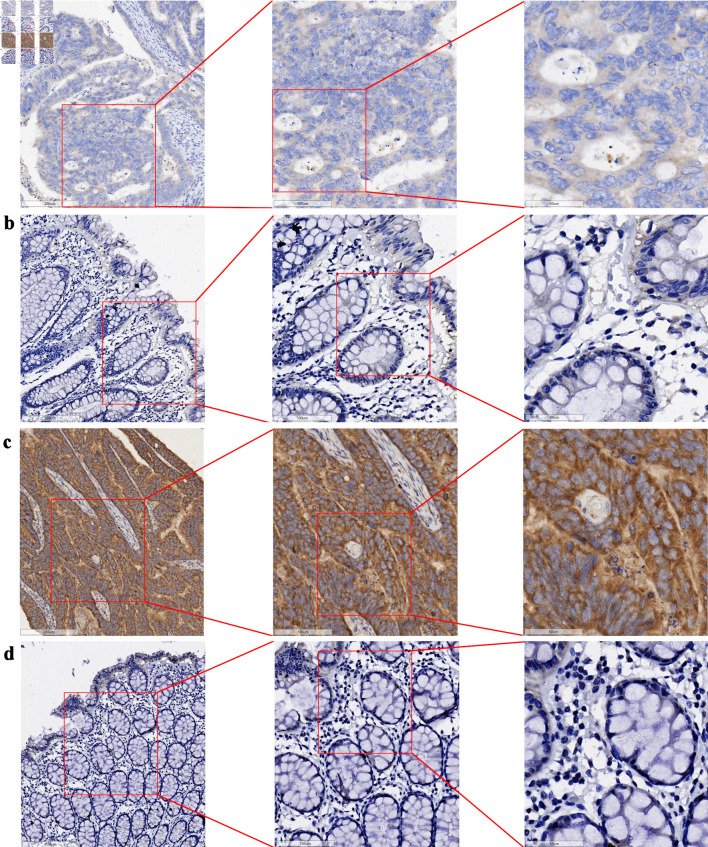
Expression of GNA15 protein in CRC tissues and their adjacent non-cancerous tissues (bar = 200 µm (left side of images), 100 µm (middle column), 50 µm (right side)). (a) CRC tissue (sample 1); (b) adjacent non-cancerous tissue (sample 1), with (a) and (b) derived from the same sample. (c) CRC tissue (sample 2); (d) adjacent non-cancerous tissue (sample 2), with (c) and (d) derived from the same sample. GNA15: G protein subunit α-15; CRC: colorectal cancer.

The tissue chips of 208 CRC and corresponding non-tumor tissue samples from Red Cross Hospital of Yulin City were scored, and the collected data were visualized with R 4.3.3. The expression level of GNA15 in tumor tissues was significantly higher than that in adjacent tissues (P = 2.74641 × 10^-85^). At the same time, the AUC value of the ROC curve was greater than 0. 99 (Fig. 1). In the immunohistochemical study, the difference in the protein expression level of GNA15 between non-cancerous adjacent tissues and CRC tissues was analyzed. The detection results of three pairs of clinical samples showed that the staining of GNA15 protein in non-cancerous tissues was significantly lighter than that in the corresponding CRC tissues (Fig. 2). These results suggest that GNA15 expression product could serve as a new screening diagnostic target.

#### UMAP analysis reveals high GNA15 expression in malignant cells compared with epithelial cells

**Figure 3:**
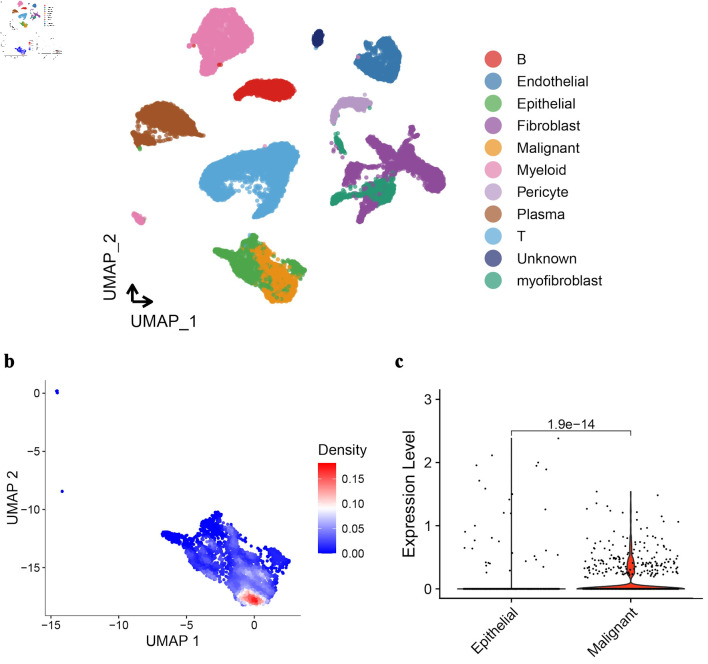
UMAP analysis and GNA15 expression in CRC tissue cells. (a) UMAP plot clusters CRC tissue cells into different types (each color represents a cell type like epithelial, malignant, fibroblast, etc.). (b) UMAP plot with a density gradient (red = high density, blue = low density) shows GNA15-expressing cell distribution. (c) Violin plot compares GNA15 expression between epithelial and malignant cells, showing significantly higher expression in malignant cells (P = 1.9 × 10^-14^). These panels demonstrate cell-type clustering in CRC via UMAP and high GNA15 expression in malignant cells. UMAP: Uniform Manifold Approximation and Projection; GNA15: G protein subunit α-15; CRC: colorectal cancer.

Cells in CRC tissue were further divided into different cell types by UMAP analysis. It can be seen that the expression profiles of malignant cells and normal epithelial cells are most similar (Fig. 3a). Among the differentially expressed genes between them, *GNA15* is clearly highly expressed in malignant cells (Fig. 3b). The violin plot of GNA15 expression in malignant cells and normal epithelial cells also shows the same result (Fig. 3c).

### B cells, CD8^+^ T cells, CD4^+^ T cells, macrophages, neutrophils and dendritic cells were all positively correlated with GNA15 expression

**Figure 4:**
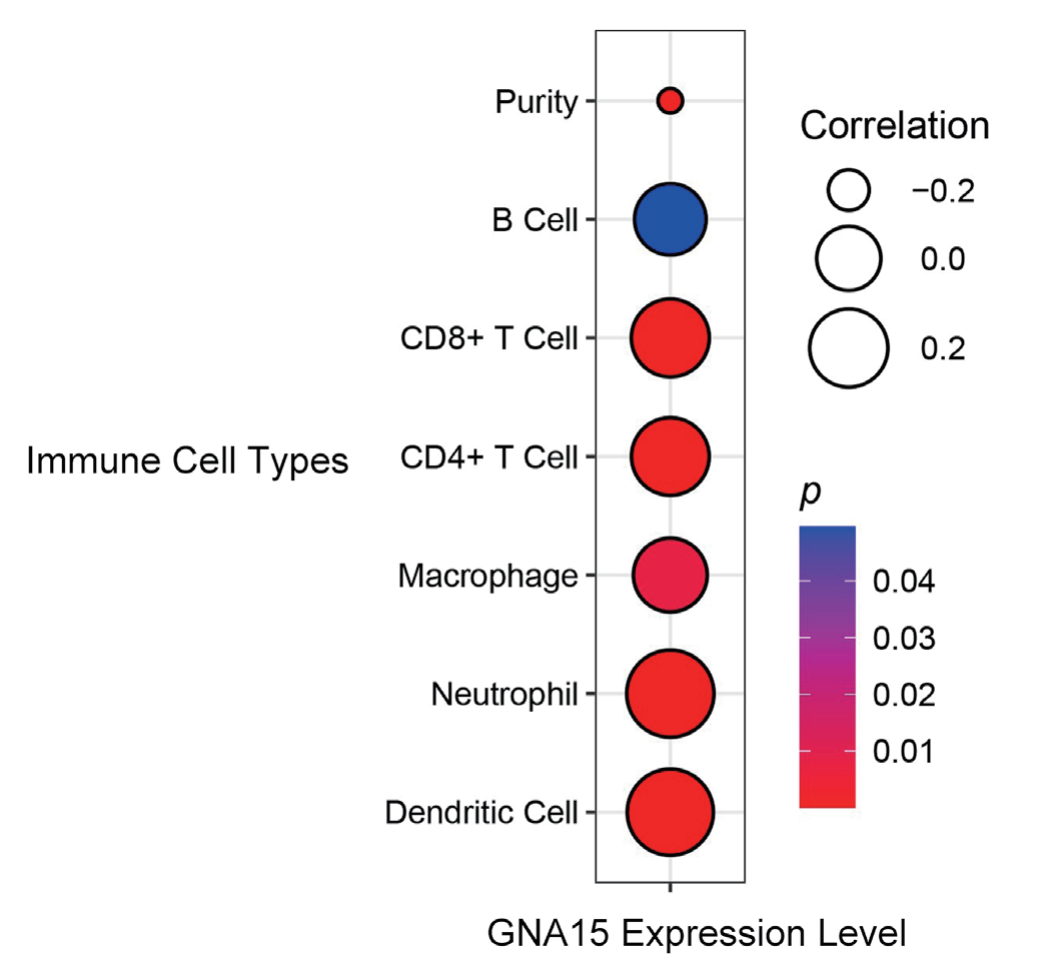
Correlation between GNA15 expression and immune cell types. Circle size indicates the correlation coefficient (larger circles indicate = stronger positive correlation, legend values: 0.2, 0.0, -0.2). Circle color represents the P value (redder = smaller P value, more significant; bluer = larger P value). Using the TIMER database, correlations were evaluated for B cell, CD8^+^ T cell, CD4^+^ T cell, macrophage, neutrophil, dendritic cell, and purity. TIMER: Tumor Immune Estimation Resource; GNA15: G protein subunit α-15.

According to the analysis of data in the TIMER database, B cells, CD8^+^ T cells, CD4^+^ T cells, macrophages, neutrophils and dendritic cells were all positively correlated with GNA15 expression, and the correlation coefficients were all greater than 0.2 except macrophages and B cells, and P < 0.05 was found in all cells except B cells (Fig. 4).

### GNA15 expression was negatively correlated with the growth of cell lines such as HCC56, DLD1

**Figure 5:**
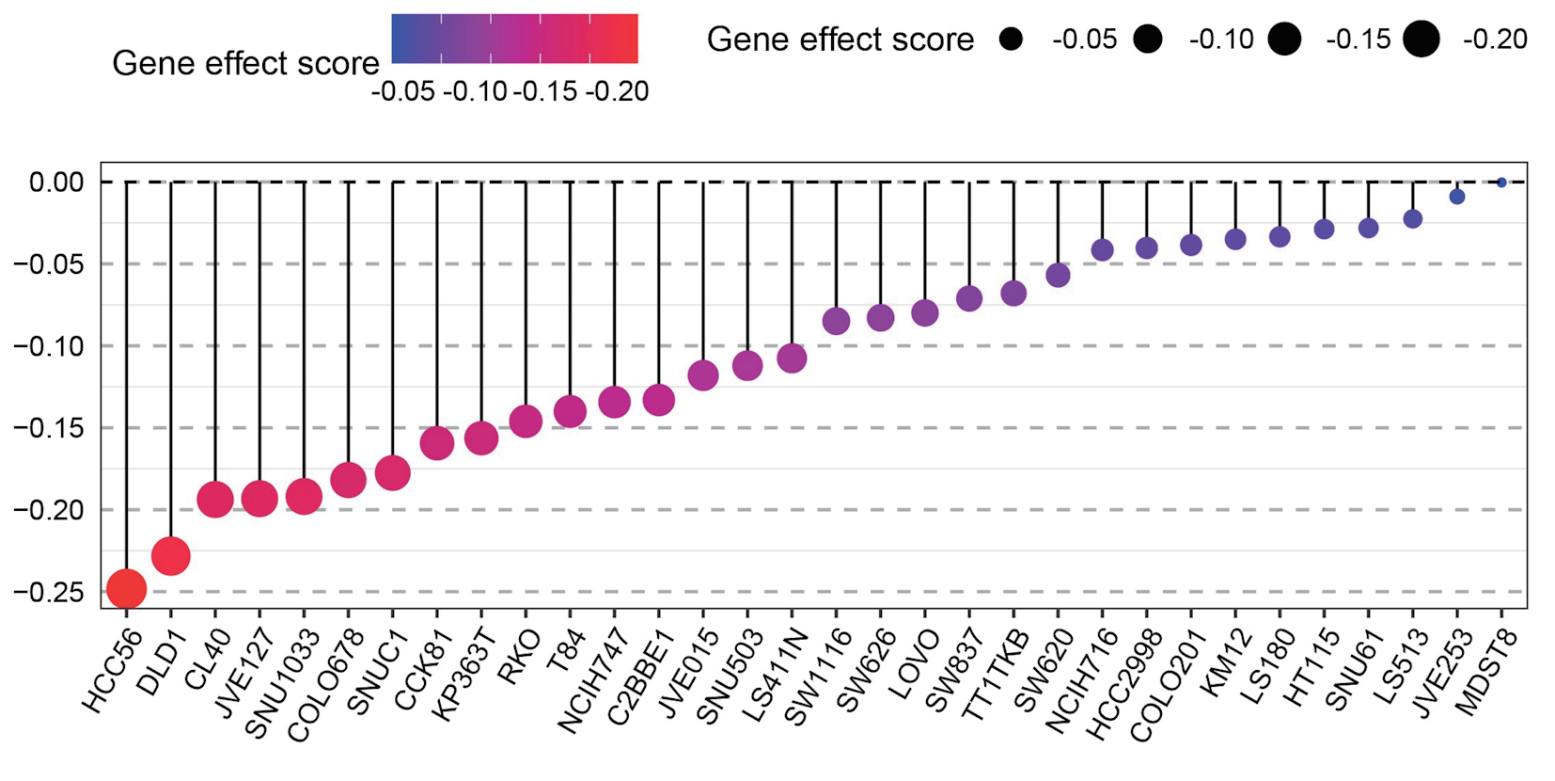
Dot plot showing the gene effect scores of different CRC cell lines (e.g., HCC56, DLD1, CL40, etc.) after *GNA15* gene knockout via CRISPR-Cas9 technology. The color gradient (from blue to red) and the size of the black dots both represent the gene effect score: a more negative score (closer to -0.20, indicated by red and larger dots) means that knocking out GNA15 has a stronger inhibitory effect on the growth of that cell line, while a score near 0 (blue, small dots) means the cell line’s growth is barely affected.

After the elimination of GNA15 using CRISPR-Cas9 gene editing technology, the growth of CRC cells was significantly interrupted. The gene effect score was calculated by CERES algorithm, and the gene effect scores of HCC56 and DLD1 cell lines showed strongly negative values (Fig. 5). It indicates that the growth of CRC cell lines such as HCC56 and DLD1 (not genes) is significantly affected after GNA15 knockout. The negative gene effect score showed that knocking out GNA15 resulted in a significant interruption of cell growth, demonstrating that GNA15 plays an important role in cell proliferation. These results suggest that GNA15 may function by influencing cell lines such as HCC56 and DLD1.

### GNA15 may act through immune-related pathways

**Figure 6:**
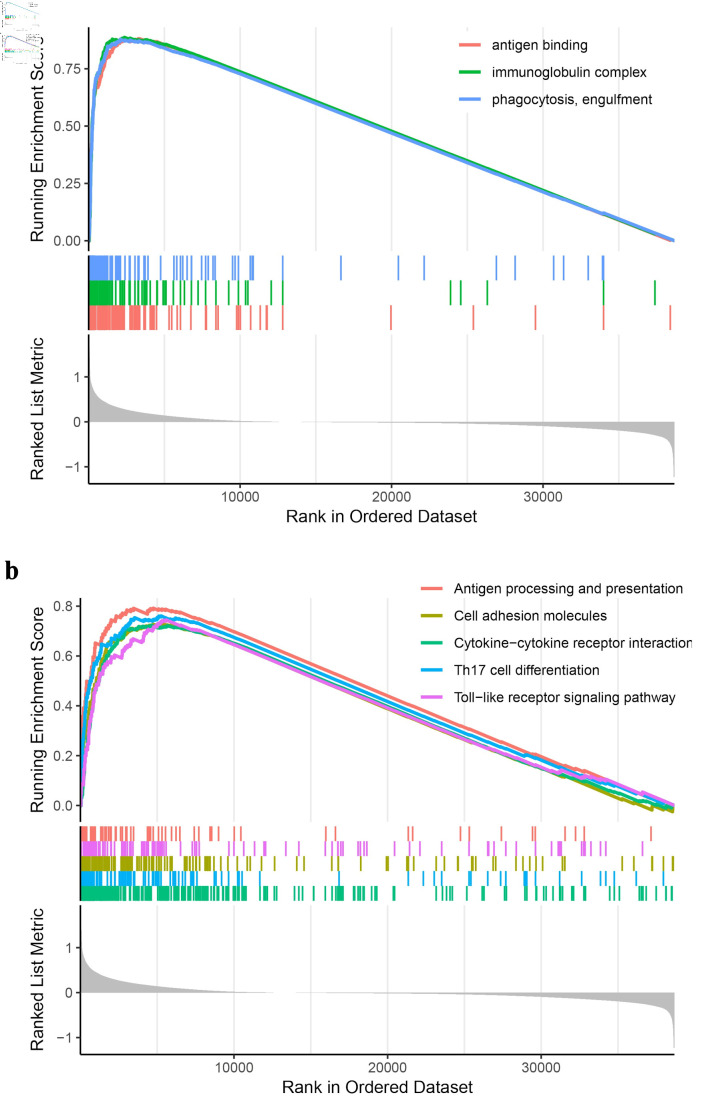
GSEA plots showing immune-related pathway enrichment in CRC. RNA sequencing data and clinical information from TCGA were analyzed using GSEA in R. (a) Enriched pathways include antigen binding, immunoglobulin complex, and phagocytosis pathways. (b) Additional enriched pathways include antigen processing/presentation, cell adhesion molecules, cytokine-cytokine receptor interaction, Th17 cell differentiation, and toll-like receptor signaling pathways. The curves and plots indicate pathways with differentially expressed genes, suggesting GNA15 may act through these immune-related pathways in CRC. GSEA: Gene Set Enrichment Analysis; CRC: colorectal cancer; TCGA: The Cancer Genome Atlas.

The RNA sequencing data of CRC and corresponding clinical information were obtained from TCGA, and the genes involved in related pathways were analyzed using the GSEA software package in R. GSEA analysis showed that differential genes were mainly concentrated in biological processes such as antigen-antibody binding, immunoglobulin complex, phagocytosis, antigen treatment presentation, cell adhesion molecules, T helper 17 (Th17) cell differentiation, and toll-like receptor signaling pathway (Fig. 6), suggesting that GNA15 may play a role through these pathways.

### Statistical analysis proves that the expression difference has statistical significance

**Figure 7:**
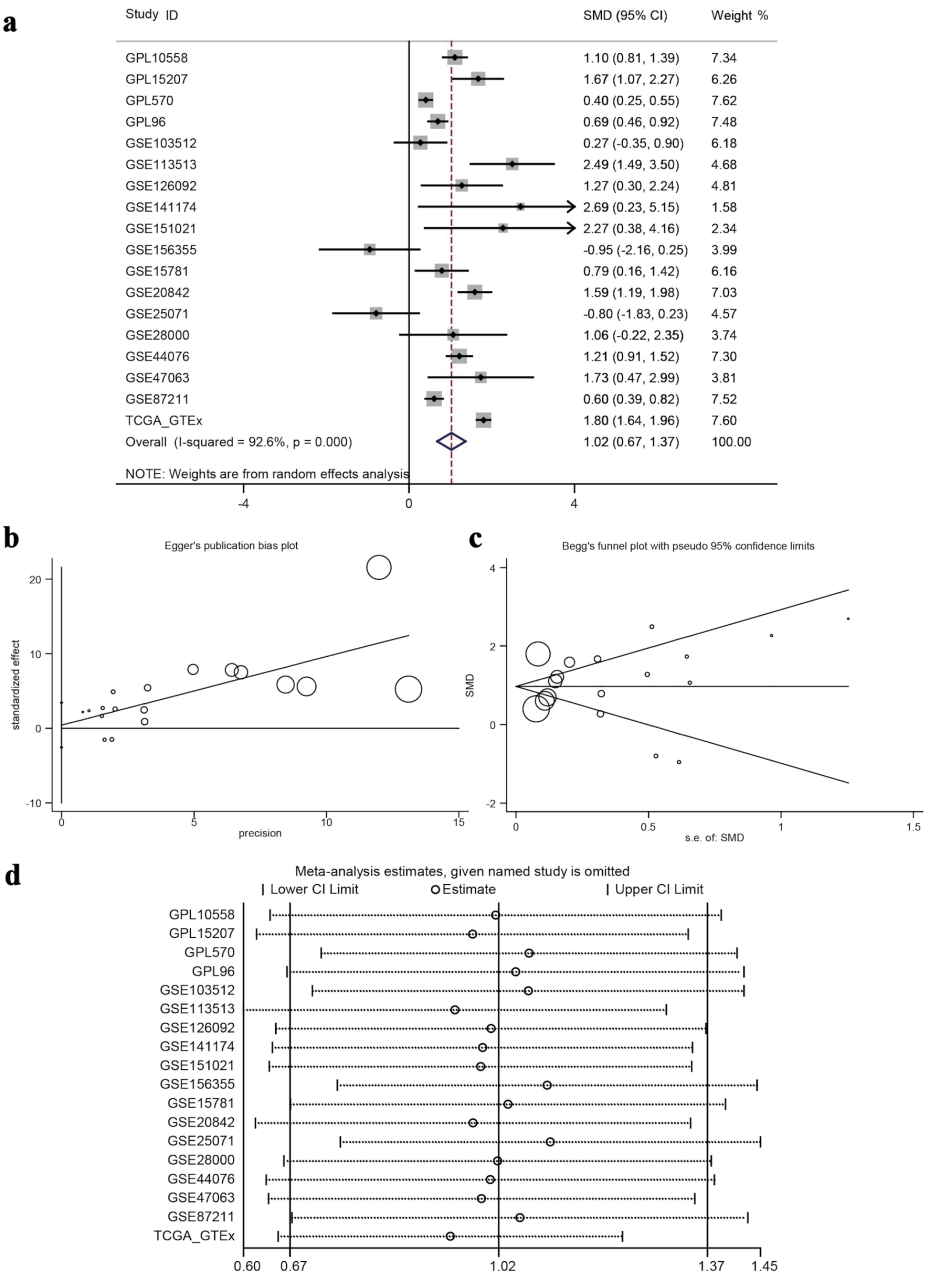
Meta-analysis and bias/sensitivity assessments. (a) A forest plot generated using inverse variance weighting is shown, where each study ID denotes a different dataset, and the diamond reflects the overall effect size with 95% CI, showing significant heterogeneity (I^2^ = 92.6%). (b) Egger’s publication bias plot, checking for the relationship between study precision and effect size to identify publication bias. (c) Begg’s funnel plot with pseudo 95% confidence limits, where symmetric distribution of study points around the mean effect suggests no significant publication bias. (d) A sensitivity analysis plot, displaying meta-analysis estimates when each study is excluded to test the robustness of the overall result. ID: identifier; GSE: Gene Set Enrichment; CRC: colorectal cancer; TCGA: The Cancer Genome Atlas; CI: confidence interval; SMD: standardized mean difference.

**Figure 8:**
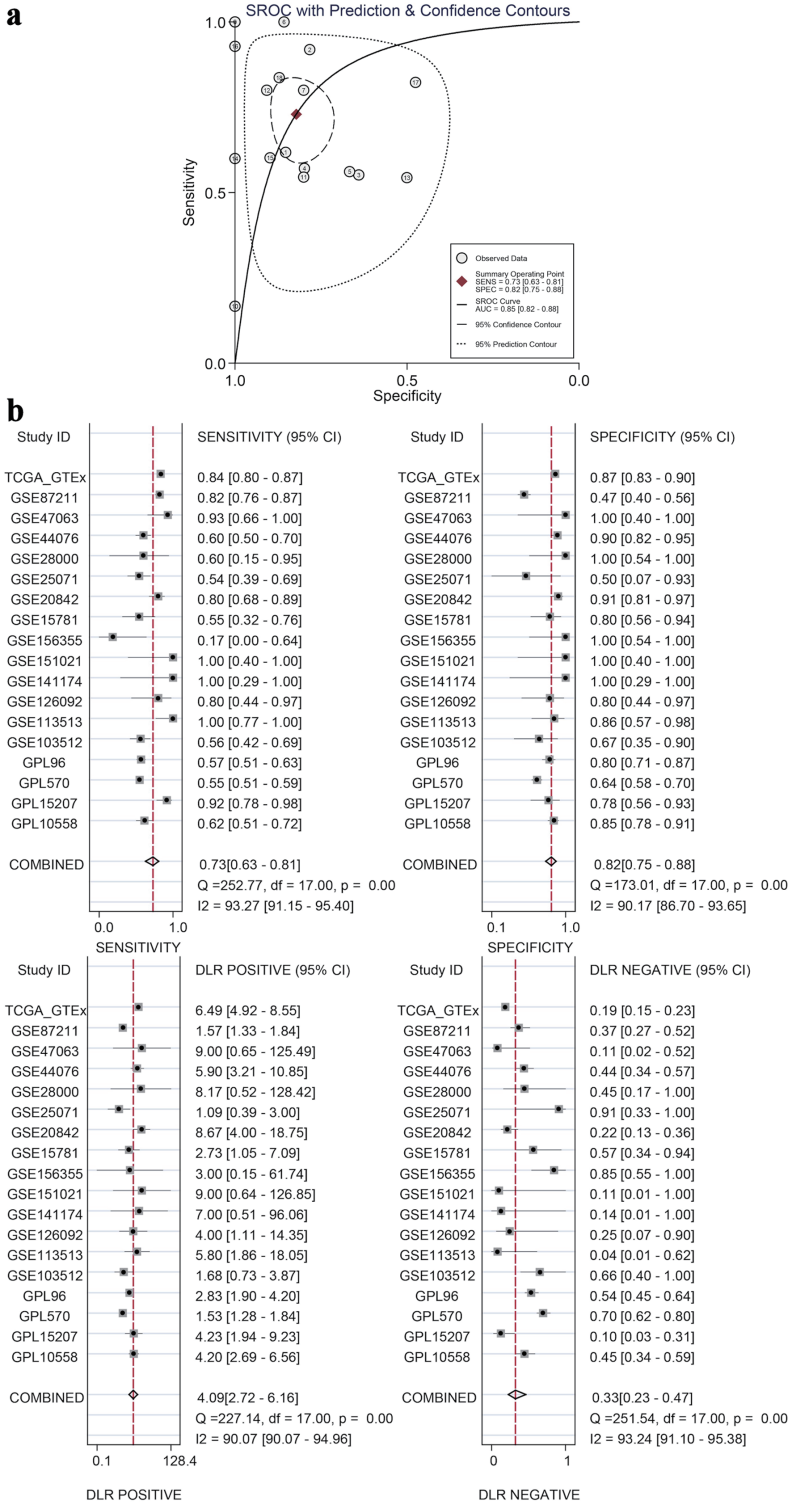
Diagnostic value by SROC and subgroup analyses. (a) An SROC curve with prediction and confidence contours, where circles are observed data, the red diamond is the summary operating point, and the AUC (0.85) indicates GNA15’s ability to distinguish cancerous and non-cancerous tissues. (b) Four subplots (sensitivity, specificity, DLR positive, and DLR negative), each with 95% CI for individual datasets and combined results, demonstrating GNA15’s high diagnostic value for CRC. SROC: summary ROC; ROC: receiver operating characteristic; DLR: diagnostic likelihood ratio; GSE: Gene Set Enrichment; CRC: colorectal cancer; TCGA: The Cancer Genome Atlas; CI: confidence interval; AUC: area under the curve.

The calculated results showed that I^2^ = 92.6% (Fig. 7a), which shows significant heterogeneity, indicating that the difference in GNA15 expression was largely attributed to non-sampling error factors. The Egger’s and Begg’s tests also showed no significant publication bias. This indicates that the conclusion of high expression of GNA15 in tumor cells is reliable (Fig. 7b, c). Also, using the pROC package in R to build the ROC and calculate the SROC curve, the AUC was 0.85, indicating that the expression level of GNA15 could better distinguish between cancerous and non-cancerous tissues (Fig. 8a). Summarizing each dataset from the TCGA and GTEx databases, the meta-analysis estimates for each dataset were relatively close, with an average of 1.02 (Fig. 7d), and the combined of sensitivity 0.73, the combined of specificity 0.82, the combined of DLR positive 4.09, and the combined of DLR negative was 0.33. These findings also show that GNA15 has high value in diagnosing CRC (Fig. 8b).

## Discussion

CRC is one of the most common malignant tumors globally, with rising incidence and mortality rates [2]. Currently, targeted therapies, alongside surgical resection, are a primary approach in CRC management. EGFR inhibitors (such as cetuximab and panitumumab) and VEGF inhibitors (such as bevacizumab) are widely used in clinical practice. However, the development of resistance and severe side effects associated with most targeted drugs remain significant challenges in CRC treatment [13]. Therefore, identifying new potential targets to enhance therapeutic efficacy and reduce adverse effects is crucial in the field of CRC.

GNA15 plays an important role in cancer-related research and plays a key role in the migration and invasion of tumor cells as well as in the malignant development of cancer, which makes it a promising therapeutic target for research. GNA15 has been shown to mediate the effects of oncogenic microRNAs (miRNAs), such as miR-211-5p, which enhance tumor cell migration and invasion by modifying the immune functions of the TME. This indicates the role of GNA15 in facilitating the transfer of metastatic capabilities between cancer cells [14]. A study identified a multi-marker panel (CEACAM6, HOXA-AS3, miR29a) with complementary roles in CRC progression [15]. CEACAM6, a cell surface glycoprotein, is significantly overexpressed in CRC tissues and correlates with tumor growth; HOXA-AS3, a long non-coding RNA, regulates cellular proliferation via epigenetic mechanisms; and miR29a, a miRNA, modulates key oncogenic pathways through post-transcriptional gene silencing. This panel highlights the value of integrating protein-coding genes, non-coding RNAs, and miRNAs for CRC diagnosis and prognosis - an approach that could be extended to GNA15. For instance, our study mentions GNA15’s mediation of miR-211-5p effects, and the newly identified miR29a may represent another regulatory miRNA interacting with GNA15, which merits further validation. In a study where CRC cells were exposed to the tobacco-specific nitrosamine 4-(methylnitrosamino)-1-(3-pyridyl)-1-butanone (NNK), a four-gene signature, including GNA15, was identified as a prognostic marker for CRC. Notably, GNA15 was found to be part of the core genes contributing to CRC cell proliferation, migration, and invasion when induced by NNK [16]. This underscores GNA15’s potential role in CRC malignancy and supports its investigation as a therapeutic target. GNA15 is of great significance in the malignant behavior of tumor cells and the development of CRC, and its in-depth study is expected to provide new directions and strategies for cancer treatment, and it is worthwhile to further explore the possibility of its use as a therapeutic target.

The results of this study have provided valuable insights into the role of GNA15 in CRC. Through the integration and analysis of multiple datasets, including immunohistochemical validation and single-cell analysis, it has been shown that GNA15 is highly expressed in CRC tissues compared to adjacent non-tumor tissues, indicating its potential as a screening diagnostic target. Noninvasive biomarkers have gained growing attention in CRC management, with circulating tumor DNA (ctDNA) leading the field. A 2025 review highlighted that advanced technologies such as droplet digital polymerase chain reaction (PCR) and next-generation sequencing (NGS) enable sensitive detection of ctDNA, which shows utility in early diagnosis, minimal residual disease (MRD) monitoring, and tracking tumor evolution [17]. Our finding that GNA15 is highly expressed in CRC tissues raises the question of whether *GNA15* mutations or copy number variations can be detected in ctDNA, which warrants future exploration. Additionally, the correlation between GNA15 expression and the TME suggests a complex interplay between the gene and immune cells in CRC progression. In the TME, B cells are closely associated with tertiary lymphoid structures (TLS), and B cells in mature TLS can differentiate into plasma cells that produce immunoglobulin (Ig)G or IgA antibodies against tumor-associated antigens, exerting an anti-tumor effect that is correlated with a better clinical outcome and immunotherapeutic response in patients [18]. Given the positive correlation between GNA15 expression and B cell infiltration, it is hypothesized that GNA15 may regulate B cell-related anti-tumor processes rather than inhibiting B-cell function. In view of the anti-tumor mechanism of B cells in CRC, the high expression of GNA15 may inhibit the development of B cells in the direction of anti-tumor function. The finding that knocking out GNA15 interrupts CRC cell growth further emphasizes its importance in cell proliferation, potentially linking its high expression to the excessive proliferation of CRC cells. This is consistent with other studies on GNA15’s role in acute myeloid leukemia cell proliferation [4], further advancing our understanding of GNA15’s mechanisms in CRC. In tumor development, antigen-antibody binding and immunoglobulin complex formation are closely related to tumor immune escape mechanisms, and the results of GNA15 in GSEA analysis suggest that GNA15 may be involved in these processes, potentially interfering with the normal immune function of the body to recognize and remove tumor cells. For example, some tumor cells may use antigen-antibody binding to escape from the immune system, and GNA15 may play a facilitating or regulating role in this process, thus affecting the development of CRC [19]. GSEA and correlation analyses provide inferential evidence for GNA15-related immune pathways, but they only reflect potential associations rather than definite causal relationships. These methods cannot fully exclude the influence of confounding factors, and further experimental verification is needed to confirm the actual regulatory mechanisms of GNA15 in these pathways.

In summary, our study provides a comprehensive evaluation of GNA15 expression and biological function in CRC, offering new insights into its role. The upregulation of GNA15 may be closely related to tumor cell proliferation and modulation of the TME, suggesting its potential application in CRC diagnosis and treatment. Notably, this study has several limitations that warrant attention. First, the single-center retrospective design and limited sample size may restrict the generalizability of GNA15’s prognostic value across diverse CRC subtypes. Second, while *in vitro* experiments validated GNA15’s pro-proliferative role, *in vivo* models recapitulating tumor-stroma interactions were lacking, which limits mechanistic insights into its microenvironment-modulating effects. Although CRISPR-Cas9 knockout experiments confirmed the role of GNA15 in CRC cell proliferation, the current study lacks rescue experiments (e.g., restoring GNA15 expression in knockout cells to verify whether cell proliferation can be recovered) and pathway-specific inhibition experiments (e.g., inhibiting key molecules in the predicted pathways to observe changes in GNA15’s regulatory effects). These experiments would help more rigorously confirm the causal relationship between GNA15 and CRC progression. Future research should further elucidate the specific functions of GNA15 within the CRC TME and its interactions with immune cells. Clinical studies are needed to validate the practical utility of GNA15 as a biomarker and explore its potential as a target for new therapeutic strategies. Overall, GNA15 not only provides a new direction for CRC research but also lays the groundwork for developing novel treatment strategies.

### Conclusions

This study comprehensively investigated GNA15 in CRC. Results showed that GNA15 is highly expressed in CRC tissues, holding potential as a screening diagnostic target. Its expression correlates with the TME, and it plays a crucial role in cell proliferation, possibly acting via immune-related pathways. Overall, this study provides new insights into GNA15’s role in CRC, suggesting its potential for CRC diagnosis and treatment. However, the single-center design and lack of *in vivo* validation limit the generalizability of our findings, and further multicenter studies are needed to confirm GNA15’s diagnostic and therapeutic value.

## Supplementary Material

Suppl 1 Clinicopathological characteristics of 208 CRC patients.

## Data Availability

The data supporting the findings of this study are available from the corresponding author upon reasonable request.
