# Supplementary material for: The Role and Mechanism of G Protein Subunit Alpha-15 in Colorectal Cancer: An Analysis of Two Hundred Eight Patient Samples and Public Datasets
Source: World J Oncol. 2025 Dec 17;17(1):116–28. doi: 10.14740/wjon2647 (PMC12758089; doi:10.14740/wjon2647)
Supplement: Suppl 1 — Clinicopathological characteristics of 208 CRC patients. [file wjon-17-01-116-s001.docx]

**Suppl 1.** Clinicopathological characteristics of 208 CRC patients

| **Point Position** | **Tissue Type** | **Result** | **Age** | **Gender** | **Macroscopic Appearance** | **Vascular Invasion** | **Neural Invasion** | **Lymph Node Invasion** | **T Stage** | **N Stage** | **M Stage** | **Survival Status** | **Clinical Stage** |
| --- | --- | --- | --- | --- | --- | --- | --- | --- | --- | --- | --- | --- | --- |
| ①A1 | Carcinoma | 6.8 | 81 | Male | Protuberant type | None | None | None | T3 | N0 | M0 | Alive | ⅡA |
| ①A2 | Pericarcinoma | 2 |  |  |  |  |  |  |  |  |  |  |  |
| ①A3 | Carcinoma | 7.6 | 55 | Female | Ulcerative type | None | None | Present | T3 | N2a | M0 | Alive | ⅢB |
| ①A4 | Pericarcinoma | 0 |  |  |  |  |  |  |  |  |  |  |  |
| ①A5 | Carcinoma | 4 | 62 | Male | Ulcerative type | None | None | None | T3 | N1c | M0 | Deceased | ⅢB |
| ①A6 | Pericarcinoma | 1.6 |  |  |  |  |  |  |  |  |  |  |  |
| ①A7 | Carcinoma | 2.8 | 57 | Female | Protuberant type | None | None | None | T3 | N0 | M0 | Alive | ⅡA |
| ①A8 | Pericarcinoma | 2.4 |  |  |  |  |  |  |  |  |  |  |  |
| ①B1 | Carcinoma | 4 | 64 | Female | Ulcerative type | None | None | None | T3 | N0 | M0 | Lost to follow-up | ⅡA |
| ①B2 | Pericarcinoma | 2.8 |  |  |  |  |  |  |  |  |  |  |  |
| ①B3 | Carcinoma | 4 | 68 | Female | Ulcerative type | None | None | None | T3 | N1c | M0 | Alive | Ⅳ期 |
| ①B4 | Pericarcinoma | 3.2 |  |  |  |  |  |  |  |  |  |  |  |
| ①B5 | Carcinoma | 6 | 39 | Male | Infiltrative type | None | None | Present | T3 | N0 | M0 | Alive | ⅡA |
| ①B6 | Pericarcinoma | 2 |  |  |  |  |  |  |  |  |  |  |  |
| ①B7 | Carcinoma | 5.6 | 64 | Male | Ulcerative type | None | None | None | T3 | N0 | M0 | Alive | ⅡA |
| ①B8 | Pericarcinoma | 0 |  |  |  |  |  |  |  |  |  |  |  |
| ①C1 | Carcinoma | 5.6 | 46 | Female | Ulcerative type | None | None | None | T3 | N0 | M0 | Alive | ⅡA |
| ①C2 | Pericarcinoma | 1.6 |  |  |  |  |  |  |  |  |  |  |  |
| ①C3 | Carcinoma | 8.8 | 64 | Male | Ulcerative type | Present | Present | Present | T3 | N1c | M0 | Alive | ⅢB |
| ①C4 | Pericarcinoma | 2 |  |  |  |  |  |  |  |  |  |  |  |
| ①C5 | Carcinoma | 7.6 | 57 | Male | Ulcerative type | None | None | Present | T3 | N0 | M0 | Lost to follow-up | ⅡA |
| ①C6 | Pericarcinoma | 2.4 |  |  |  |  |  |  |  |  |  |  |  |
| ①C7 | Carcinoma | 4 | 70 | Male | Ulcerative type | None | None | Present | T3 | N1a | M0 | Alive | ⅢB |
| ①C8 | Pericarcinoma | 2.4 |  |  |  |  |  |  |  |  |  |  |  |
| ①D1 | Carcinoma | 4 | 65 | Male | Protuberant type | None | None | None | T4a | N0 | M0 | Deceased | ⅡB |
| ①D2 | Pericarcinoma | 3.2 |  |  |  |  |  |  |  |  |  |  |  |
| ①D3 | Carcinoma | 6.8 | 68 | Male | Infiltrative type | None | None | Present | T3 | N1c | M0 | Alive | ⅢB |
| ①D4 | Pericarcinoma | 2.4 |  |  |  |  |  |  |  |  |  |  |  |
| ①D5 | Carcinoma | 6.8 | 86 | Male | Ulcerative type | Present | None | Present | T3 | N1a | M0 | Deceased | ⅢB |
| ①D6 | Pericarcinoma | 3.2 |  |  |  |  |  |  |  |  |  |  |  |
| ①D7 | Carcinoma | 4 | 55 | Female | Ulcerative type | None | None | None | T3 | N0 | M0 | Alive | ⅡA |
| ①D8 | Pericarcinoma | 1.6 |  |  |  |  |  |  |  |  |  |  |  |
| ①E1 | Carcinoma | 6.4 | 72 | Male | Protuberant type | None | None | None | T3 | N0 | M0 | Alive | ⅡA |
| ①E2 | Pericarcinoma | 2.4 |  |  |  |  |  |  |  |  |  |  |  |
| ①E3 | Carcinoma | 12 | 69 | Male | Protuberant type | None | None | None | T2 | N0 | M0 | Alive | I |
| ①E4 | Pericarcinoma | 3.2 |  |  |  |  |  |  |  |  |  |  |  |
| ①E5 | Carcinoma | 4.6 | 57 | Male | Protuberant type | None | None | None | T3 | N1c | M0 | Alive | ⅢB |
| ①E6 | Pericarcinoma | 0 |  |  |  |  |  |  |  |  |  |  |  |
| ①E7 | Carcinoma | 1.6 | 76 | Female | Ulcerative type | None | None | None | T3 | N0 | M0 | Alive | ⅡA |
| ①E8 | Pericarcinoma | 0 |  |  |  |  |  |  |  |  |  |  |  |
| ①F1 | Carcinoma | 10.8 | 44 | Male | Ulcerative type | Present | Present | Present | T3 | N2a | M0 | Alive | ⅢB |
| ①F2 | Pericarcinoma | 3.2 |  |  |  |  |  |  |  |  |  |  |  |
| ①F3 | Carcinoma | 9.2 | 51 | Male | Ulcerative type | None | None | Present | T3 | N1 | M0 | Alive | ⅡA |
| ①F4 | Pericarcinoma | 2.4 |  |  |  |  |  |  |  |  |  |  |  |
| ①F5 | Carcinoma | 6.4 | 80 | Male | Ulcerative type | None | None | None | T3 | N1c | M0 | Deceased | ⅢB |
| ①F6 | Pericarcinoma | 1.6 |  |  |  |  |  |  |  |  |  |  |  |
| ①F7 | Carcinoma | 4 | 45 | Male | Ulcerative type | None | None | Present | T3 | N1b | M0 | Alive | ⅢB |
| ①F8 | Pericarcinoma | 0.8 |  |  |  |  |  |  |  |  |  |  |  |
| ②A1 | Carcinoma | 4 | 74 | Male | Ulcerative type | Present | None | Present | T3 | N1b | M0 | Deceased | ⅢB |
| ②A2 | Pericarcinoma | 0 |  |  |  |  |  |  |  |  |  |  |  |
| ②A3 | Carcinoma | 4 | 71 | Male | Protuberant type | None | None | Present | T3 | N2a | M0 | Alive | ⅢB |
| ②A4 | Pericarcinoma | 0 |  |  |  |  |  |  |  |  |  |  |  |
| ②A5 | Carcinoma | 4 | 66 | Female | Ulcerative type | None | Present | None | T3 | N0 | M0 | Alive | ⅡA |
| ②A6 | Pericarcinoma | 0 |  |  |  |  |  |  |  |  |  |  |  |
| ②A7 | Carcinoma | 4 | 55 | Female | Ulcerative type | None | None | Present | T3 | N1b | M0 | Alive | ⅢB |
| ②A8 | Pericarcinoma | 0 |  |  |  |  |  |  |  |  |  |  |  |
| ②B1 | Carcinoma | 4 | 65 | Female | Ulcerative type | None | None | None | T3 | N0 | M0 | Alive | ⅡA |
| ②B2 | Pericarcinoma | 0 |  |  |  |  |  |  |  |  |  |  |  |
| ②B3 | Carcinoma | 4 | 60 | Female | Ulcerative type | None | None | None | T2 | N0 | M0 | Alive | Ⅰ |
| ②B4 | Pericarcinoma | 0 |  |  |  |  |  |  |  |  |  |  |  |
| ②B5 | Carcinoma | 4 | 81 | Male | Ulcerative type | None | None | None | T3 | N0 | M0 | Alive | ⅡA |
| ②B6 | Pericarcinoma | 0 |  |  |  |  |  |  |  |  |  |  |  |
| ②B7 | Carcinoma | 4 | 67 | Male | Protuberant type | Present | None | None | T3 | N0 | M0 | Alive | ⅡA |
| ②B8 | Pericarcinoma | 0 |  |  |  |  |  |  |  |  |  |  |  |
| ②C1 | Carcinoma | 4 | 49 | Female | Protuberant type | None | None | None | T3 | N1c | M0 | Deceased | ⅢB |
| ②C2 | Pericarcinoma | 0 |  |  |  |  |  |  |  |  |  |  |  |
| ②C3 | Carcinoma | 4 | 72 | Female | Infiltrative type | None | None | None | T3 | N0 | M0 | Alive | ⅡA |
| ②C4 | Pericarcinoma | 0 |  |  |  |  |  |  |  |  |  |  |  |
| ②C5 | Carcinoma | 4 | 52 | Female | Protuberant type | None | None | None | T3 | N0 | M0 | Alive | ⅡA |
| ②C6 | Pericarcinoma | 0 |  |  |  |  |  |  |  |  |  |  |  |
| ②C7 | Carcinoma | 4 | 56 | Male | Protuberant type | None | None | Present | T3 | N2a | M0 | Alive | ⅢB |
| ②C8 | Pericarcinoma | 0 |  |  |  |  |  |  |  |  |  |  |  |
| ②D1 | Carcinoma | 4 | 90 | Male | Protuberant type | None | None | None | T3 | N0 | M0 | Deceased | ⅡA |
| ②D2 | Pericarcinoma | 0 |  |  |  |  |  |  |  |  |  |  |  |
| ②D3 | Carcinoma | 4 | 77 | Male | Protuberant type | None | None | None | T3 | N0 | M0 | Deceased | ⅡA |
| ②D4 | Pericarcinoma | 0 |  |  |  |  |  |  |  |  |  |  |  |
| ②D5 | Carcinoma | 4 | 70 | Male | Protuberant type | Present | None | None | T2 | N0 | M0 | Alive | Ⅰ |
| ②D6 | Pericarcinoma | 0 |  |  |  |  |  |  |  |  |  |  |  |
| ②D7 | Carcinoma | 4 | 31 | Male | Ulcerative type | Present | Present | Present | T3 | N2a | M0 | Alive | ⅢB |
| ②D8 | Pericarcinoma | 0 |  |  |  |  |  |  |  |  |  |  |  |
| ②E1 | Carcinoma | 4 | 70 | Male | Protuberant type | None | None | Present | T3 | N2a | M0 | Alive | ⅢB |
| ②E2 | Pericarcinoma | 0 |  |  |  |  |  |  |  |  |  |  |  |
| ②E3 | Carcinoma | 4 | 56 | Female | Ulcerative type | None | None | Present | T3 | N1a | M0 | Alive | ⅢB |
| ②E4 | Pericarcinoma | 0 |  |  |  |  |  |  |  |  |  |  |  |
| ②E5 | Carcinoma | 4 | 73 | Female | Protuberant type | Present | None | None | T3 | N0 | M0 | Alive | ⅡA |
| ②E6 | Pericarcinoma | 0 |  |  |  |  |  |  |  |  |  |  |  |
| ②E7 | Carcinoma | 4 | 81 | Male | Ulcerative type | None | None | None | T3 | N0 | M0 | Alive | ⅡA |
| ②E8 | Pericarcinoma | 0 |  |  |  |  |  |  |  |  |  |  |  |
| ②F1 | Carcinoma | 4 | 62 | Male | Ulcerative type | None | None | None | T3 | N0 | M0 | Deceased | ⅡA |
| ②F2 | Pericarcinoma | 0 |  |  |  |  |  |  |  |  |  |  |  |
| ②F3 | Carcinoma | 4 | 15 | Female | Protuberant type | None | None | Present | T3 | N1b | M0 | Alive | ⅢB |
| ②F4 | Pericarcinoma | 0 |  |  |  |  |  |  |  |  |  |  |  |
| ②F5 | Carcinoma | 4 | 27 | Male | Protuberant type | None | None | None | T3 | N1a | M0 | Alive | ⅢB |
| ②F6 | Pericarcinoma | 0 |  |  |  |  |  |  |  |  |  |  |  |
| ②F7 | Carcinoma | 4 | 61 | Male | Ulcerative type | None | None | None | T3 | N0 | M0 | Alive | ⅡA |
| ②F8 | Pericarcinoma | 0 |  |  |  |  |  |  |  |  |  |  |  |
| ③A1 | Carcinoma | 4 | 68 | Male | Ulcerative type | Present | None | Present | T3 | N2b | M0 | Alive | ⅢB |
| ③A2 | Pericarcinoma | 0 |  |  |  |  |  |  |  |  |  |  |  |
| ③A3 | Carcinoma | 4 | 56 | Female | Ulcerative type | None | None | None | T3 | N0 | M0 | Alive | ⅡA |
| ③A4 | Pericarcinoma | 0 |  |  |  |  |  |  |  |  |  |  |  |
| ③A5 | Carcinoma | 4 | 52 | Male | Ulcerative type | None | None | None | T3 | Nx | M0 | Deceased | ⅡA |
| ③A6 | Pericarcinoma | 0 |  |  |  |  |  |  |  |  |  |  |  |
| ③A7 | Carcinoma | 4 | 50 | Female | Protuberant type | None | None | None | T3 | N0 | M0 | Alive | ⅡA |
| ③A8 | Pericarcinoma | 0 |  |  |  |  |  |  |  |  |  |  |  |
| ③B1 | Carcinoma | 4 | 63 | Male | Ulcerative type | None | None | None | T3 | N1b | M0 | Alive | ⅢB |
| ③B2 | Pericarcinoma | 0 |  |  |  |  |  |  |  |  |  |  |  |
| ③B3 | Carcinoma | 4 | 76 | Female | Ulcerative type | None | None | None | T3 | N0 | M0 | Alive | ⅡA |
| ③B4 | Pericarcinoma | 0 |  |  |  |  |  |  |  |  |  |  |  |
| ③B5 | Carcinoma | 4 | 80 | Male | Ulcerative type | None | Present | None | T3 | N0 | M0 | Alive | ⅡA |
| ③B6 | Pericarcinoma | 0 |  |  |  |  |  |  |  |  |  |  |  |
| ③B7 | Carcinoma | 4 | 56 | Male | Protuberant type | None | None | None | T3 | N0 | M0 | Alive | ⅡA |
| ③B8 | Pericarcinoma | 0 |  |  |  |  |  |  |  |  |  |  |  |
| ③C1 | Carcinoma | 4 | 63 | Female | Protuberant type | None | None | None | T3 | N0 | M0 | Alive | ⅡA |
| ③C2 | Pericarcinoma | 0 |  |  |  |  |  |  |  |  |  |  |  |
| ③C3 | Carcinoma | 4 | 52 | Female | Ulcerative type | None | None | None | T3 | N0 | M0 | Alive | ⅡA |
| ③C4 | Pericarcinoma | 0 |  |  |  |  |  |  |  |  |  |  |  |
| ③C5 | Carcinoma | 4 | 55 | Male | Ulcerative type | Present | None | None | T3 | N0 | M0 | Alive | ⅡA |
| ③C6 | Pericarcinoma | 0 |  |  |  |  |  |  |  |  |  |  |  |
| ③C7 | Carcinoma | 4 | 74 | Male | Ulcerative type | None | None | None | T3 | N0 | M0 | Deceased | ⅡA |
| ③C8 | Pericarcinoma | 0 |  |  |  |  |  |  |  |  |  |  |  |
| ③D1 | Carcinoma | 4 | 56 | Male | Ulcerative type | None | Present | Present | T3 | N1b | M0 | Alive | ⅢB |
| ③D2 | Pericarcinoma | 0 |  |  |  |  |  |  |  |  |  |  |  |
| ③D3 | Carcinoma | 4 | 63 | Male | Infiltrative type | Present | Present | Present | T3 | N1b | M0 | Alive | ⅢB |
| ③D4 | Pericarcinoma | 0 |  |  |  |  |  |  |  |  |  |  |  |
| ③D5 | Carcinoma | 4 | 72 | Female | Ulcerative type | None | None | None | T3 | N0 | M0 | Alive | ⅡA |
| ③D6 | Pericarcinoma | 0 |  |  |  |  |  |  |  |  |  |  |  |
| ③D7 | Carcinoma | 4 | 63 | Male | Ulcerative type | None | None | None | T3 | N0 | M0 | Alive | ⅡA |
| ③D8 | Pericarcinoma | 0 |  |  |  |  |  |  |  |  |  |  |  |
| ③E1 | Carcinoma | 4 | 68 | Female | Ulcerative type | None | None | Present | T3 | N1c | M0 | Alive | ⅢB |
| ③E2 | Pericarcinoma | 0 |  |  |  |  |  |  |  |  |  |  |  |
| ③E3 | Carcinoma | 4 | 64 | Female | Ulcerative type | None | None | Present | T3 | N1b | M0 | Deceased | ⅢB |
| ③E4 | Pericarcinoma | 0 |  |  |  |  |  |  |  |  |  |  |  |
| ③E5 | Carcinoma | 4 | 56 | Male | Ulcerative type | None | None | None | T3 | N0 | M0 | Alive | ⅡA |
| ③E6 | Pericarcinoma | 0 |  |  |  |  |  |  |  |  |  |  |  |
| ③E7 | Carcinoma | 4 | 70 | Male | Ulcerative type | Present | None | Present | T2 | N1a | M0 | Alive | ⅢB |
| ③E8 | Pericarcinoma | 0 |  |  |  |  |  |  |  |  |  |  |  |
| ③F1 | Carcinoma | 4 | 63 | Female | Ulcerative type | Present | Present | None | T3 | N0 | M0 | Alive | ⅡA |
| ③F2 | Pericarcinoma | 0 |  |  |  |  |  |  |  |  |  |  |  |
| ③F3 | Carcinoma | 4 | 64 | Male | Ulcerative type | None | None | None | T3 | N0 | M0 | Alive | ⅡA |
| ③F4 | Pericarcinoma | 0 |  |  |  |  |  |  |  |  |  |  |  |
| ③F5 | Carcinoma | 4 | 82 | Female | Ulcerative type | None | Present | Present | T3 | N1a | M0 | Deceased | ⅢB |
| ③F6 | Pericarcinoma | 0 |  |  |  |  |  |  |  |  |  |  |  |
| ③F7 | Carcinoma | 4 | 70 | Female | Ulcerative type | None | None | None | T3 | N0 | M0 | Alive | ⅡA |
| ③F8 | Pericarcinoma | 0 |  |  |  |  |  |  |  |  |  |  |  |
| ④A1 | Carcinoma | 4 | 92 | Female | Ulcerative type | None | Present | Present | T4a | N2a | M0 | Alive | ⅢB |
| ④A2 | Pericarcinoma | 0 |  |  |  |  |  |  |  |  |  |  |  |
| ④A3 | Carcinoma | 4 | 75 | Female | Ulcerative type | None | None | None | T3 | N0 | M0 | Alive | ⅡA |
| ④A4 | Pericarcinoma | 0 |  |  |  |  |  |  |  |  |  |  |  |
| ④A5 | Carcinoma | 4 | 74 | Female | Ulcerative type | None | None | None | T3 | N0 | M0 | Alive | ⅡA |
| ④A6 | Pericarcinoma | 0 |  |  |  |  |  |  |  |  |  |  |  |
| ④B1 | Carcinoma | 4 | 77 | Female | Ulcerative type | None | None | None | T3 | N0 | M0 | Alive | ⅡA |
| ④B2 | Pericarcinoma | 0 |  |  |  |  |  |  |  |  |  |  |  |
| ④B3 | Carcinoma | 4 | 66 | Female | Protuberant type | None | None | Present | T3 | N0 | M0 | Alive | ⅡA |
| ④B4 | Pericarcinoma | 0 |  |  |  |  |  |  |  |  |  |  |  |
| ④B5 | Carcinoma | 4 | 65 | Female | Protuberant type | None | None | None | T3 | N0 | M0 | Alive | ⅡA |
| ④B6 | Pericarcinoma | 0 |  |  |  |  |  |  |  |  |  |  |  |
| ④C1 | Carcinoma | 4 | 59 | Male | Ulcerative type | None | None | Present | T3 | N1b | M0 | Alive | ⅢB |
| ④C2 | Pericarcinoma | 0 |  |  |  |  |  |  |  |  |  |  |  |
| ④C3 | Carcinoma | 4 | 87 | Male | Protuberant type | Present | None | Present | T3 | N2b | M0 | Alive | ⅢB |
| ④C4 | Pericarcinoma | 0 |  |  |  |  |  |  |  |  |  |  |  |
| ④C5 | Carcinoma | 4 | 57 | Male | Protuberant type | None | None | None | T3 | N0 | M0 | Alive | ⅡA |
| ④C6 | Pericarcinoma | 0 |  |  |  |  |  |  |  |  |  |  |  |
| ④C7 | Carcinoma | 4 | 64 | Male | Ulcerative type | None | None | None | T3 | N0 | M0 | Alive | ⅡA |
| ④B7 | Pericarcinoma | 0 |  |  |  |  |  |  |  |  |  |  |  |
| ④D1 | Carcinoma | 4 | 70 | Female | Ulcerative type | None | Present | None | T3 | N1c | M0 | Deceased | ⅢB |
| ④D2 | Pericarcinoma | 0 |  |  |  |  |  |  |  |  |  |  |  |
| ④D3 | Carcinoma | 4 | 62 | Male | Ulcerative type | None | None | Present | T3 | N2a | M0 | Alive | ⅢB |
| ④D4 | Pericarcinoma | 0 |  |  |  |  |  |  |  |  |  |  |  |
| ④D5 | Carcinoma | 4 | 67 | Male | Ulcerative type | None | None | Present | T4a | N1a | M0 | Alive | ⅢC |
| ④D6 | Pericarcinoma | 0 |  |  |  |  |  |  |  |  |  |  |  |
| ④E1 | Carcinoma | 4 | 77 | Male | Ulcerative type | None | None | Present | T3 | N1a | M0 | Alive | ⅢB |
| ④E2 | Pericarcinoma | 0 |  |  |  |  |  |  |  |  |  |  |  |
| ④E3 | Carcinoma | 4 | 79 | Male | Protuberant type | None | None | None | T3 | N0 | M0 | Alive | ⅡA |
| ④E4 | Pericarcinoma | 0 |  |  |  |  |  |  |  |  |  |  |  |
| ④E5 | Carcinoma | 4 | 80 | Male | Protuberant type | None | None | None | T2 | N0 | M0 | Alive | ⅡA |
| ④E6 | Pericarcinoma | 0 |  |  |  |  |  |  |  |  |  |  |  |
| ⑤A1 | Carcinoma | 4 | 57 | Female | Protuberant type | None | None | None | T3 | N0 | M0 | Alive | ⅡA |
| ⑤A2 | Pericarcinoma | 0 |  |  |  |  |  |  |  |  |  |  |  |
| ⑤A3 | Carcinoma | 4 | 67 | Female | Ulcerative type | None | None | None | T3 | N1c/x | M0 | Lost to follow-up | ⅢB |
| ⑤A4 | Pericarcinoma | 0 |  |  |  |  |  |  |  |  |  |  |  |
| ⑤A5 | Carcinoma | 4 | 77 | Male | Infiltrative type | None | None | None | T3 | N0 | M0 | Alive | ⅡA |
| ⑤A6 | Pericarcinoma | 0 |  |  |  |  |  |  |  |  |  |  |  |
| ⑤B1 | Carcinoma | 4 | 64 | Female | Ulcerative type | None | None | None | T3 | N0 | M0 | Alive | ⅡA |
| ⑤B2 | Pericarcinoma | 0 |  |  |  |  |  |  |  |  |  |  |  |
| ⑤B3 | Carcinoma | 4 | 57 | Female | Ulcerative type | None | None | Present | T2 | N1c | M0 | Alive | ⅢA |
| ⑤B4 | Pericarcinoma | 0 |  |  |  |  |  |  |  |  |  |  |  |
| ⑤B5 | Carcinoma | 4 | 62 | Male | Protuberant type | None | None | None | T3 | N0 | M0 | Alive | ⅡA |
| ⑤B6 | Pericarcinoma | 0 |  |  |  |  |  |  |  |  |  |  |  |
| ⑤C1 | Carcinoma | 4 | 55 | Female | Ulcerative type | None | None | None | T3 | N1a | M0 | Alive | ⅢB |
| ⑤C2 | Pericarcinoma | 0 |  |  |  |  |  |  |  |  |  |  |  |
| ⑤C3 | Carcinoma | 4 | 63 | Male | Protuberant type | Present | None | Present | T3 | N1a | M0 | Deceased | ⅢB |
| ⑤C4 | Pericarcinoma | 0 |  |  |  |  |  |  |  |  |  |  |  |
| ⑤C5 | Carcinoma | 4 | 66 | Male | Ulcerative type | None | None | Present | T2 | N1b | M0 | Alive | ⅢA |
| ⑤C6 | Pericarcinoma | 0 |  |  |  |  |  |  |  |  |  |  |  |
| ⑤D1 | Carcinoma | 4 | 81 | Male | Ulcerative type | Present | None | None | T3 | N0 | M0 | Lost to follow-up | ⅡA |
| ⑤D2 | Pericarcinoma | 0 |  |  |  |  |  |  |  |  |  |  |  |
| ⑤D3 | Carcinoma | 4 | 25 | Male | Ulcerative type | None | None | None | T3 | N0 | M0 | Alive | ⅡA |
| ⑤D4 | Pericarcinoma | 0 |  |  |  |  |  |  |  |  |  |  |  |
| ⑤D5 | Carcinoma | 4 | 68 | Male | Ulcerative type | None | Present | None | T3 | N0 | M0 | Alive | ⅡA |
| ⑤D6 | Pericarcinoma | 0 |  |  |  |  |  |  |  |  |  |  |  |
| ⑤E1 | Carcinoma | 4 | 52 | Male | Ulcerative type | None | None | None | T3 | N0 | M0 | Alive | ⅡA |
| ⑤E2 | Pericarcinoma | 0 |  |  |  |  |  |  |  |  |  |  |  |
| ⑤E3 | Carcinoma | 4 | 57 | Female | Ulcerative type | None | None | None | T2 | N0 | M0 | Alive | Ⅰ |
| ⑤E4 | Pericarcinoma | 0 |  |  |  |  |  |  |  |  |  |  |  |
| ⑤E5 | Carcinoma | 4 | 74 | Male | Ulcerative type | None | None | None | T2 | N0 | M0 | Alive | ⅠV |
| ⑤E6 | Pericarcinoma | 0 |  |  |  |  |  |  |  |  |  |  |  |
| ⑥A1 | Carcinoma | 4 | 58 | Male | Protuberant type | None | None | Present | T3 | N1b | M0 | Alive | ⅢB |
| ⑥A2 | Pericarcinoma | 0 |  |  |  |  |  |  |  |  |  |  |  |
| ⑥A3 | Carcinoma | 4 | 49 | Male | Infiltrative type | Present | None | None | T3 | Nx | M0 | Deceased | ⅡA |
| ⑥A4 | Pericarcinoma | 0 |  |  |  |  |  |  |  |  |  |  |  |
| ⑥A5 | Carcinoma | 4 | 73 | Male | Ulcerative type | None | None | None | T3 | N0 | M0 | Alive | ⅡA |
| ⑥A6 | Pericarcinoma | 0 |  |  |  |  |  |  |  |  |  |  |  |
| ⑥B1 | Carcinoma | 4 | 63 | Female | Protuberant type | None | None | None | T3 | N1c/x | M0 | Deceased | ⅢB |
| ⑥B2 | Pericarcinoma | 0 |  |  |  |  |  |  |  |  |  |  |  |
| ⑥B3 | Carcinoma | 4 | 83 | Male | Ulcerative type | Present | None | Present | T4a | N1b | M0 | Deceased | ⅢC |
| ⑥B4 | Pericarcinoma | 0 |  |  |  |  |  |  |  |  |  |  |  |
| ⑥B5 | Carcinoma | 4 | 72 | Male | Protuberant type | None | None | None | T2 | N0 | M0 | Alive | Ⅰ |
| ⑥B6 | Pericarcinoma | 0 |  |  |  |  |  |  |  |  |  |  |  |
| ⑥C1 | Carcinoma | 4 | 69 | Male | Protuberant type | None | None | None | T3 | N0 | M0 | Alive | ⅡA |
| ⑥C2 | Pericarcinoma | 0 |  |  |  |  |  |  |  |  |  |  |  |
| ⑥C3 | Carcinoma | 4 | 44 | Female | Ulcerative type | None | None | None | T3 | N0 | M0 | Alive | ⅡA |
| ⑥C4 | Pericarcinoma | 0 |  |  |  |  |  |  |  |  |  |  |  |
| ⑥C5 | Carcinoma | 4 | 75 | Male | Ulcerative type | None | Present | None | T3 | N0 | M0 | Alive | ⅡA |
| ⑥C6 | Pericarcinoma | 0 |  |  |  |  |  |  |  |  |  |  |  |
| ⑥D1 | Carcinoma | 4 | 67 | Male | Protuberant type | None | None | None | T3 | N0 | M0 | Alive | ⅡA |
| ⑥D2 | Pericarcinoma | 0 |  |  |  |  |  |  |  |  |  |  |  |
| ⑥D3 | Carcinoma | 4 | 59 | Male | Protuberant type | Present | None | Present | T4b | N2b | M0 | Deceased | ⅢC |
| ⑥D4 | Pericarcinoma | 0 |  |  |  |  |  |  |  |  |  |  |  |
| ⑥D5 | Carcinoma | 4 | 46 | Male | Ulcerative type | None | None | None | T3 | N0 | M0 | Lost to follow-up | ⅡA |
| ⑥D6 | Pericarcinoma | 0 |  |  |  |  |  |  |  |  |  |  |  |
| ⑥E1 | Carcinoma | 4 | 46 | Male | Protuberant type | None | None | None | T3 | N0 | M0 | Alive | ⅡA |
| ⑥E2 | Pericarcinoma | 0 |  |  |  |  |  |  |  |  |  |  |  |
| ⑥E3 | Carcinoma | 4 | 69 | Female | Protuberant type | None | None | None | T2 | N1a | M0 | Alive | ⅢA |
| ⑥E4 | Pericarcinoma | 0 |  |  |  |  |  |  |  |  |  |  |  |
| ⑥E5 | Carcinoma | 4 | 55 | Female | Ulcerative type | None | None | Present | T3 | N1b | M0 | Lost to follow-up | ⅢB |
| ⑥E6 | Pericarcinoma | 0 |  |  |  |  |  |  |  |  |  |  |  |
| ⑦A1 | Carcinoma | 4 | 32 | Male | Ulcerative type | Present | Present | None | T3 | Nx | M0 | Deceased | ⅡA |
| ⑦A2 | Pericarcinoma | 0 |  |  |  |  |  |  |  |  |  |  |  |
| ⑦A3 | Carcinoma | 4 | 58 | Male | Ulcerative type | None | None | None | T3 | N0 | M0 | Alive | ⅡA |
| ⑦A4 | Pericarcinoma | 0 |  |  |  |  |  |  |  |  |  |  |  |
| ⑦A5 | Carcinoma | 4 | 61 | Female | Ulcerative type | None | None | Present | T3 | N1a | M0 | Deceased | ⅢB |
| ⑦A6 | Pericarcinoma | 0 |  |  |  |  |  |  |  |  |  |  |  |
| ⑦B1 | Carcinoma | 4 | 44 | Female | Protuberant type | Present | None | Present | T3 | N2a | M0 | Deceased | ⅢB |
| ⑦B2 | Pericarcinoma | 0 |  |  |  |  |  |  |  |  |  |  |  |
| ⑦B3 | Carcinoma | 4 | 46 | Female | Protuberant type | None | None | Present | T2 | N1a | M0 | Alive | ⅢA |
| ⑦B4 | Pericarcinoma | 0 |  |  |  |  |  |  |  |  |  |  |  |
| ⑦B5 | Carcinoma | 4 | 62 | Female | Protuberant type | None | None | None | Tis | N0 | M0 | Alive | 0 |
| ⑦B6 | Pericarcinoma | 0 |  |  |  |  |  |  |  |  |  |  |  |
| ⑦C1 | Carcinoma | 4 | 50 | Female | Ulcerative type | None | None | None | T2 | N0 | M0 | Alive | Ⅰ |
| ⑦C2 | Pericarcinoma | 0 |  |  |  |  |  |  |  |  |  |  |  |
| ⑦C3 | Carcinoma | 4 | 68 | Female | Ulcerative type | None | None | None | T2 | N0 | M0 | Alive | Ⅰ |
| ⑦C4 | Pericarcinoma | 0 |  |  |  |  |  |  |  |  |  |  |  |
| ⑦C5 | Carcinoma | 4 | 67 | Male | Protuberant type | None | None | Present | T3 | N1b | M0 | Alive | ⅢB |
| ⑦C6 | Pericarcinoma | 0 |  |  |  |  |  |  |  |  |  |  |  |
| ⑦D1 | Carcinoma | 4 | 56 | Female | Protuberant type | None | None | None | T2 | N0 | M0 | Lost to follow-up | Ⅰ |
| ⑦D2 | Pericarcinoma | 0 |  |  |  |  |  |  |  |  |  |  |  |
| ⑦D3 | Carcinoma | 4 | 64 | Male | Ulcerative type | None | None | Present | T3 | N1a | M0 | Alive | ⅢB |
| ⑦D4 | Pericarcinoma | 0 |  |  |  |  |  |  |  |  |  |  |  |
| ⑦D5 | Carcinoma | 4 | 66 | Female | Ulcerative type | None | None | None | T3 | Nx | M0 | Alive | ⅡA |
| ⑦D6 | Pericarcinoma | 0 |  |  |  |  |  |  |  |  |  |  |  |
| ⑦E1 | Carcinoma | 4 | 54 | Male | Ulcerative type | None | None | Present | T3 | N0 | M0 | Deceased | ⅡA |
| ⑦E2 | Pericarcinoma | 0 |  |  |  |  |  |  |  |  |  |  |  |
| ⑦E3 | Carcinoma | 4 | 71 | Male | Ulcerative type | Present | None | Present | T3 | N1b | M0 | Alive | ⅢB |
| ⑦E4 | Pericarcinoma | 0 |  |  |  |  |  |  |  |  |  |  |  |
| ⑦E5 | Carcinoma | 4 | 64 | Male | Ulcerative type | None | None | None | T3 | N1a | M0 | Deceased | ⅢB |
| ⑦E6 | Pericarcinoma | 0 |  |  |  |  |  |  |  |  |  |  |  |
| ⑧A1 | Carcinoma | 4 | 47 | Female | Ulcerative type | None | None | Present | T3 | N1b | M0 | Lost to follow-up | ⅢB |
| ⑧A2 | Pericarcinoma | 0 |  |  |  |  |  |  |  |  |  |  |  |
| ⑧A3 | Carcinoma | 4 | 76 | Male | Ulcerative type | Present | None | None | T3 | N0 | M0 | Alive | ⅡA |
| ⑧A4 | Pericarcinoma | 0 |  |  |  |  |  |  |  |  |  |  |  |
| ⑧A5 | Carcinoma | 4 | 47 | Male | Ulcerative type | None | None | None | T3 | N0 | M0 | Alive | ⅡA |
| ⑧A6 | Pericarcinoma | 0 |  |  |  |  |  |  |  |  |  |  |  |
| ⑧B1 | Carcinoma | 4 | 64 | Female | Ulcerative type | None | None | Present | T3 | N2b | M0 | Alive | ⅢB |
| ⑧B2 | Pericarcinoma | 0 |  |  |  |  |  |  |  |  |  |  |  |
| ⑧B3 | Carcinoma | 4 | 70 | Female | Ulcerative type | None | None | None | T3 | N0 | M0 | Alive | ⅡA |
| ⑧B4 | Pericarcinoma | 0 |  |  |  |  |  |  |  |  |  |  |  |
| ⑧B5 | Carcinoma | 4 | 78 | Male | Ulcerative type | None | None | None | T3 | N0 | M0 | Alive | ⅡA |
| ⑧B6 | Pericarcinoma | 0 |  |  |  |  |  |  |  |  |  |  |  |
| ⑧C1 | Carcinoma | 4 | 70 | Male | Ulcerative type | None | None | None | T3 | N0 | M0 | Alive | ⅡA |
| ⑧C2 | Pericarcinoma | 0 |  |  |  |  |  |  |  |  |  |  |  |
| ⑧C3 | Carcinoma | 4 | 71 | Male | Ulcerative type | None | None | None | T3 | N0 | M0 | Alive | ⅡA |
| ⑧C4 | Pericarcinoma | 0 |  |  |  |  |  |  |  |  |  |  |  |
| ⑧C5 | Carcinoma | 4 | 68 | Female | Protuberant type | None | None | None | T3 | N0 | M0 | Alive | ⅡA |
| ⑧C6 | Pericarcinoma | 0 |  |  |  |  |  |  |  |  |  |  |  |
| ⑧D1 | Carcinoma | 4 | 52 | Male | Protuberant type | None | None | Present | T4a | N1c | M0 | Deceased | ⅢB |
| ⑧D2 | Pericarcinoma | 0 |  |  |  |  |  |  |  |  |  |  |  |
| ⑧D3 | Carcinoma | 4 | 76 | Female | Ulcerative type | None | None | Present | T3 | N1c | M0 | Alive | ⅢB |
| ⑧D4 | Pericarcinoma | 0 |  |  |  |  |  |  |  |  |  |  |  |
| ⑧D5 | Carcinoma | 4 | 56 | Male | Protuberant type | None | None | None | T2 | N0 | M0 | Alive | ⅡA |
| ⑧D6 | Pericarcinoma | 0 |  |  |  |  |  |  |  |  |  |  |  |
| ⑧E1 | Carcinoma | 4 | 63 | Male | Ulcerative type | None | None | None | T3 | Nx | M0 | Alive | ⅡA |
| ⑧E2 | Pericarcinoma | 0 |  |  |  |  |  |  |  |  |  |  |  |
| ⑧E3 | Carcinoma | 4 | 76 | Male | Ulcerative type | None | None | None | T3 | N0 | M0 | Alive | ⅡA |
| ⑧E4 | Pericarcinoma | 0 |  |  |  |  |  |  |  |  |  |  |  |
| ⑧E5 | Carcinoma | 4 | 57 | Male | Ulcerative type | None | None | None | T3 | N2a | M0 | Alive | ⅢB |
| ⑧E6 | Pericarcinoma | 0 |  |  |  |  |  |  |  |  |  |  |  |
| ⑨A1 | Carcinoma | 4 | 82 | Female | Ulcerative type | None | None | None | T3 | N0 | M0 | Alive | ⅡA |
| ⑨A2 | Pericarcinoma | 0 |  |  |  |  |  |  |  |  |  |  |  |
| ⑨A3 | Carcinoma | 4 | 57 | Female | Ulcerative type | None | None | None | T3 | N0 | M0 | Alive | ⅡA |
| ⑨A4 | Pericarcinoma | 0 |  |  |  |  |  |  |  |  |  |  |  |
| ⑨A5 | Carcinoma | 4 | 55 | Female | Infiltrative type | Present | Present | Present | T3 | N1c | M0 | Alive | ⅢB |
| ⑨A6 | Pericarcinoma | 0 |  |  |  |  |  |  |  |  |  |  |  |
| ⑨B1 | Carcinoma | 4 | 74 | Female | Protuberant type | None | None | None | T2 | N0 | M0 | Alive | ⅡA |
| ⑨B2 | Pericarcinoma | 0 |  |  |  |  |  |  |  |  |  |  |  |
| ⑨B3 | Carcinoma | 4 | 47 | Male | Ulcerative type | None | None | None | T3 | N0 | M0 | Alive | ⅡA |
| ⑨B4 | Pericarcinoma | 0 |  |  |  |  |  |  |  |  |  |  |  |
| ⑨B5 | Carcinoma | 4 | 54 | Female | Protuberant type | None | None | None | T3 | N0 | M0 | Alive | ⅡA |
| ⑨B6 | Pericarcinoma | 0 |  |  |  |  |  |  |  |  |  |  |  |
| ⑨C1 | Carcinoma | 4 | 79 | Female | Ulcerative type | None | None | None | T3 | Nx | M0 | Deceased | ⅡA |
| ⑨C2 | Pericarcinoma | 0 |  |  |  |  |  |  |  |  |  |  |  |
| ⑨C3 | Carcinoma | 4 | 46 | Female | Ulcerative type | None | None | None | T3 | N0 | M0 | Alive | ⅡA |
| ⑨C4 | Pericarcinoma | 0 |  |  |  |  |  |  |  |  |  |  |  |
| ⑨C5 | Carcinoma | 4 | 79 | Male | Ulcerative type | None | None | None | T3 | N0 | M0 | Deceased | ⅡA |
| ⑨C6 | Pericarcinoma | 0 |  |  |  |  |  |  |  |  |  |  |  |
| ⑨D1 | Carcinoma | 4 | 64 | Female | Ulcerative type | None | None | None | T3 | N0 | M0 | Alive | ⅡA |
| ⑨D2 | Pericarcinoma | 0 |  |  |  |  |  |  |  |  |  |  |  |
| ⑨D3 | Carcinoma | 4 | 43 | Male | Ulcerative type | None | None | None | T3 | N2a | M0 | Alive | ⅢB |
| ⑨D4 | Pericarcinoma | 0 |  |  |  |  |  |  |  |  |  |  |  |
| ⑨D5 | Carcinoma | 4 | 73 | Male | Ulcerative type | None | None | None | T3 | N0 | M0 | Alive | ⅡA |
| ⑨D6 | Pericarcinoma | 0 |  |  |  |  |  |  |  |  |  |  |  |
| ⑨E1 | Carcinoma | 4 | 59 | Male | Ulcerative type | None | None | None | T3 | N0 | M0 | Alive | ⅡA |
| ⑨E2 | Pericarcinoma | 0 |  |  |  |  |  |  |  |  |  |  |  |
| ⑨E3 | Carcinoma | 4 | 60 | Male | Ulcerative type | None | None | None | T3 | N0 | M0 | Alive | ⅡA |
| ⑨E4 | Pericarcinoma | 0 |  |  |  |  |  |  |  |  |  |  |  |
| ⑨E5 | Carcinoma | 4 | 76 | Female | Ulcerative type | None | None | None | T3 | N0 | M0 | Alive | ⅡA |
| ⑨E6 | Pericarcinoma | 0 |  |  |  |  |  |  |  |  |  |  |  |
| ⑩A1 | Carcinoma | 4 | 83 | Male | Ulcerative type | None | None | None | T3 | N0 | M0 | Alive | ⅡA |
| ⑩A2 | Pericarcinoma | 0 |  |  |  |  |  |  |  |  |  |  |  |
| ⑩A3 | Carcinoma | 4 | 66 | Female | Ulcerative type | None | None | Present | T3 | N1a | M0 | Deceased | ⅢB |
| ⑩A4 | Pericarcinoma | 0 |  |  |  |  |  |  |  |  |  |  |  |
| ⑩A5 | Carcinoma | 4 | 67 | Male | Infiltrative type | None | Present | None | T3 | N1c | M0 | Alive | ⅢB |
| ⑩A6 | Pericarcinoma | 0 |  |  |  |  |  |  |  |  |  |  |  |
| ⑩B1 | Carcinoma | 4 | 65 | Female | Ulcerative type | None | None | None | T3 | N1c | M0 | Alive | ⅢB |
| ⑩B2 | Pericarcinoma | 0 |  |  |  |  |  |  |  |  |  |  |  |
| ⑩B3 | Carcinoma | 4 | 44 | Male | Ulcerative type | None | Present | None | T3 | N0 | M0 | Alive | ⅡA |
| ⑩B4 | Pericarcinoma | 0 |  |  |  |  |  |  |  |  |  |  |  |
| ⑩B5 | Carcinoma | 4 | 73 | Male | Ulcerative type | None | None | None | T3 | N1c | M0 | Alive | ⅢB |
| ⑩B6 | Pericarcinoma | 0 |  |  |  |  |  |  |  |  |  |  |  |
| ⑩C1 | Carcinoma | 4 | 68 | Male | Ulcerative type | Present | Present | Present | T3 | N2b | M0 | Alive | ⅢB |
| ⑩C2 | Pericarcinoma | 0 |  |  |  |  |  |  |  |  |  |  |  |
| ⑩C3 | Carcinoma | 4 | 63 | Female | Ulcerative type | None | None | None | T3 | N0 | M0 | Lost to follow-up | ⅡA |
| ⑩C4 | Pericarcinoma | 0 |  |  |  |  |  |  |  |  |  |  |  |
| ⑩C5 | Carcinoma | 4 | 63 | Female | Ulcerative type | None | None | None | T3 | N0 | M0 | Alive | ⅡA |
| ⑩C6 | Pericarcinoma | 0 |  |  |  |  |  |  |  |  |  |  |  |
| ⑩D1 | Carcinoma | 4 | 57 | Male | Ulcerative type | Present | None | None | T3 | N0 | M0 | Alive | ⅡA |
| ⑩D2 | Pericarcinoma | 0 |  |  |  |  |  |  |  |  |  |  |  |
| ⑩D3 | Carcinoma | 4 | 54 | Female | Ulcerative type | None | None | None | T3 | N1a | M0 | Lost to follow-up | ⅢB |
| ⑩D4 | Pericarcinoma | 0 |  |  |  |  |  |  |  |  |  |  |  |
| ⑩D5 | Carcinoma | 4 | 74 | Female | Ulcerative type | None | None | None | T3 | N0 | M0 | Alive | ⅡA |
| ⑩D6 | Pericarcinoma | 0 |  |  |  |  |  |  |  |  |  |  |  |
| ⑩E1 | Carcinoma | 4 | 47 | Male | Protuberant type | None | None | Present | T3 | N1c | M0 | Alive | ⅢB |
| ⑩E2 | Pericarcinoma | 0 |  |  |  |  |  |  |  |  |  |  |  |
| ⑩E3 | Carcinoma | 4 | 52 | Male | Ulcerative type | None | Present | None | T3 | N2a | M0 | Alive | ⅢB |
| ⑩E4 | Pericarcinoma | 0 |  |  |  |  |  |  |  |  |  |  |  |
| ⑩E5 | Carcinoma | 4 | 56 | Female | Ulcerative type | None | None | Present | T3 | N1c | M0 | Deceased | ⅢB |
| ⑩E6 | Pericarcinoma | 0 |  |  |  |  |  |  |  |  |  |  |  |
| ⑪A1 | Carcinoma | 4 | 63 | Male | Ulcerative type | Present | None | None | T3 | N0 | M0 | Alive | ⅡA |
| ⑪A2 | Pericarcinoma | 0 |  |  |  |  |  |  |  |  |  |  |  |
| ⑪A3 | Carcinoma | 4 | 83 | Female | Ulcerative type | Present | None | Present | T4b | N1a | M0 | Deceased | ⅢB |
| ⑪A4 | Pericarcinoma | 0 |  |  |  |  |  |  |  |  |  |  |  |
| ⑪A5 | Carcinoma | 4 | 66 | Male | Ulcerative type | None | None | None | T3 | N0 | M0 | Alive | ⅡA |
| ⑪A6 | Pericarcinoma | 0 |  |  |  |  |  |  |  |  |  |  |  |
| ⑪B1 | Carcinoma | 4 | 85 | Female | Ulcerative type | None | None | None | T3 | N0 | M0 | Alive | ⅡA |
| ⑪B2 | Pericarcinoma | 0 |  |  |  |  |  |  |  |  |  |  |  |
| ⑪B3 | Carcinoma | 4 | 63 | Male | Ulcerative type | Present | None | None | T3 | N0 | M0 | Lost to follow-up | ⅡA |
| ⑪B4 | Pericarcinoma | 0 |  |  |  |  |  |  |  |  |  |  |  |
| ⑪B5 | Carcinoma | 4 | 80 | Female | Ulcerative type | None | None | None | T3 | N0 | M0 | Deceased | ⅡA |
| ⑪B6 | Pericarcinoma | 0 |  |  |  |  |  |  |  |  |  |  |  |
| ⑪C1 | Carcinoma | 4 | 78 | Male | Protuberant type | None | None | None | Tis | N0 | M0 | Alive | 0 |
| ⑪C2 | Pericarcinoma | 0 |  |  |  |  |  |  |  |  |  |  |  |
| ⑪C3 | Carcinoma | 4 | 86 | Female | Ulcerative type | None | None | None | T3 | N0 | M0 | Deceased | ⅡA |
| ⑪C4 | Pericarcinoma | 0 |  |  |  |  |  |  |  |  |  |  |  |
| ⑪C5 | Carcinoma | 4 | 67 | Male | Ulcerative type | None | None | None | T3 | N0 | M0 | Alive | ⅡA |
| ⑪C6 | Pericarcinoma | 0 |  |  |  |  |  |  |  |  |  |  |  |
| ⑪D1 | Carcinoma | 4 | 69 | Male | Ulcerative type | None | Present | None | T3 | N0 | M0 | Alive | ⅡA |
| ⑪D2 | Pericarcinoma | 0 |  |  |  |  |  |  |  |  |  |  |  |
| ⑪D3 | Carcinoma | 4 | 37 | Male | Infiltrative type | None | None | None | T3 | N0 | M0 | Alive | ⅡA |
| ⑪D4 | Pericarcinoma | 0 |  |  |  |  |  |  |  |  |  |  |  |
| ⑪D5 | Carcinoma | 4 | 56 | Male | Ulcerative type | None | None | None | T3 | N0 | M0 | Alive | ⅡA |
| ⑪D6 | Pericarcinoma | 0 |  |  |  |  |  |  |  |  |  |  |  |
| ⑪E1 | Carcinoma | 4 | 45 | Male | Ulcerative type | None | None | None | T3 | Nx | M0 | Alive | ⅡA |
| ⑪E2 | Pericarcinoma | 0 |  |  |  |  |  |  |  |  |  |  |  |
| ⑪E3 | Carcinoma | 4 | 59 | Male | Ulcerative type | Present | None | None | T3 | N0 | M0 | Alive | ⅡA |
| ⑪E4 | Pericarcinoma | 0 |  |  |  |  |  |  |  |  |  |  |  |
| ⑪E5 | Carcinoma | 4 | 57 | Female | Ulcerative type | None | None | Present | T3 | N2a | M0 | Alive | ⅢB |
| ⑪E6 | Pericarcinoma | 0 |  |  |  |  |  |  |  |  |  |  |  |
| ⑫A1 | Carcinoma | 4 | 47 | Female | Protuberant type | None | None | None | Tis | N0 | M0 | Alive | 0 |
| ⑫A2 | Pericarcinoma | 0 |  |  |  |  |  |  |  |  |  |  |  |
| ⑫A3 | Carcinoma | 4 | 71 | Male | Infiltrative type | None | Present | None | T3 | N0 | M0 | Deceased | ⅡA |
| ⑫A4 | Pericarcinoma | 0 |  |  |  |  |  |  |  |  |  |  |  |
| ⑫A5 | Carcinoma | 4 | 54 | Female | Protuberant type | None | None | None | T3 | Nx | M0 | Alive | ⅡA |
| ⑫A6 | Pericarcinoma | 0 |  |  |  |  |  |  |  |  |  |  |  |
| ⑫B1 | Carcinoma | 4 | 44 | Male | Ulcerative type | Present | None | Present | T3 | N1a | M0 | Alive | ⅢB |
| ⑫B2 | Pericarcinoma | 0 |  |  |  |  |  |  |  |  |  |  |  |
| ⑫B3 | Carcinoma | 4 | 67 | Female | Ulcerative type | None | None | Present | T3 | N1c | M0 | Alive | ⅢB |
| ⑫B4 | Pericarcinoma | 0 |  |  |  |  |  |  |  |  |  |  |  |
| ⑫B5 | Carcinoma | 4 | 77 | Male | Ulcerative type | Present | None | Present | T3 | N1a | M0 | Alive | ⅢB |
| ⑫B6 | Pericarcinoma | 0 |  |  |  |  |  |  |  |  |  |  |  |
| ⑫C1 | Carcinoma | 4 | 47 | Male | Protuberant type | Present | None | None | T3 | N2b | M0 | Alive | ⅢB |
| ⑫C2 | Pericarcinoma | 0 |  |  |  |  |  |  |  |  |  |  |  |
| ⑫C3 | Carcinoma | 4 | 65 | Male | Ulcerative type | None | None | None | T3 | N0 | M0 | Alive | ⅡA |
| ⑫C4 | Pericarcinoma | 0 |  |  |  |  |  |  |  |  |  |  |  |
| ⑫C5 | Carcinoma | 4 | 79 | Male | Ulcerative type | None | None | None | T3 | N0 | M0 | Alive | ⅡA |
| ⑫C6 | Pericarcinoma | 0 |  |  |  |  |  |  |  |  |  |  |  |
| ⑫D1 | Carcinoma | 4 | 73 | Male | Ulcerative type | None | None | None | T3 | N0 | M0 | Alive | ⅡA |
| ⑫D2 | Pericarcinoma | 0 |  |  |  |  |  |  |  |  |  |  |  |
| ⑫D3 | Carcinoma | 4 | 87 | Female | Ulcerative type | Present | None | Present | T3 | N2a | M0 | Alive | ⅢB |
| ⑫D4 | Pericarcinoma | 0 |  |  |  |  |  |  |  |  |  |  |  |
| ⑫D5 | Carcinoma | 4 | 80 | Male | Ulcerative type | Present | None | None | T3 | N0 | M0 | Alive | ⅡA |
| ⑫D6 | Pericarcinoma | 0 |  |  |  |  |  |  |  |  |  |  |  |
| ⑫E1 | Carcinoma | 4 | 72 | Male | Ulcerative type | None | None | Present | T3 | N2a | M0 | Alive | ⅢB |
| ⑫E2 | Pericarcinoma | 0 |  |  |  |  |  |  |  |  |  |  |  |
| ⑫E3 | Carcinoma | 4 | 56 | Female | Protuberant type | Present | None | Present | T3 | N1a | M0 | Alive | ⅢB |
| ⑫E4 | Pericarcinoma | 0 |  |  |  |  |  |  |  |  |  |  |  |
| ⑫E5 | Carcinoma | 4 | 57 | Female | Ulcerative type | Present | Present | Present | T3 | N2b | M0 | Alive | ⅢB |
| ⑫E6 | Pericarcinoma | 0 |  |  |  |  |  |  |  |  |  |  |  |
